# Ceramides and neuroinflammation as immunometabolic drivers and biomarkers of major depressive disorder, treatment-resistant depression, and suicidal vulnerability

**DOI:** 10.3389/fphar.2026.1805884

**Published:** 2026-04-14

**Authors:** Martin G. Garcia-Juarez, Blanca E. Alvarez-Salas, Ali F. Ruiz-Higareda, Iván A. Marino-Martinez, Antonio A. Perez-Maya

**Affiliations:** 1 Departamento de Microbiología, Facultad de Medicina, Universidad Autónoma de Nuevo León, Monterrey, Nuevo León, Mexico; 2 Departamento de Bioquímica y Medicina Molecular, Facultad de Medicina, Universidad Autónoma de Nuevo León, Monterrey, Nuevo León, Mexico; 3 Departamento de Patología, Facultad de Medicina, Universidad Autónoma de Nuevo León, Monterrey, Nuevo León, Mexico

**Keywords:** blood-brain barrier, ceramides, lipid biomarkers, major depressive disorder, neuroinflammation, suicidal behavior, treatment-resistant depression (TRD)

## Abstract

Major depressive disorder (MDD) and treatment-resistant depression (TRD) are biologically heterogeneous conditions with substantial suicide risk, yet current diagnostic frameworks lack validated biological markers for patient stratification. This narrative review examines the role of ceramides and lipid metabolism as immunometabolic drivers and potential biomarkers in MDD, TRD, and suicidal vulnerability. We integrate evidence from lipidomic, neuroinflammatory, and translational studies to characterize how ceramides, generated through *de novo* synthesis, sphingomyelinase-mediated pathways, and salvage mechanisms, participate in microglial priming, blood-brain barrier compromise, synaptic dysfunction, and regulated cell death. Ceramide accumulation, modulated by HPA axis dysregulation, adiposity, and comorbid metabolic conditions, intersects with tryptophan-kynurenine pathway alterations and mitochondrial bioenergetic deficits, converging on a multi-level immunometabolic framework relevant to depressive and treatment-resistant phenotypes. Circulating ceramide species, particularly C16–C24:1, show consistent elevations in MDD and correlate with symptom severity, antidepressant exposure, and sex-specific patterns, while indirect evidence links lipid dysregulation to suicidal behavior. Acid sphingomyelinase inhibition by functional antidepressants highlights a pharmacologically relevant axis. Current evidence is constrained by cross-sectional designs, small samples, and heterogeneous platforms. Longitudinal, multi-omic studies with harmonized protocols are needed to determine whether ceramide profiles can inform risk stratification and personalized interventions in precision psychiatry.

## 1 Introduction

Major depressive disorder (MDD), the most prevalent condition within the broader category of depressive disorders, constitutes one of the leading causes of disability worldwide, in Low- and Middle-Income Countries (LMICs) represented the 80.19% of cases of disability-adjusted life years (DALYs) (Yu et al., 2025). The studies to date indicate that from 1990 to 2021 the estimated of Depressive disorders as a group affected an estimated 332 million people globally (Tang et al., 2025). However, as of the time of this review, no global data are available for the year 2025.

MDD represents a markedly heterogeneous clinical entity in which affective, cognitive, and somatic symptoms converge, with significant impact on individual and social functioning (Christensen et al., 2020) Current diagnostic systems, such as the DSM and ICD, describe depression based on symptomatic and temporal criteria that enable clinical identification but are limited in their capacity to capture the biological complexity and diversity of the disorder’s developmental trajectories. This phenomenological reduction contributes to the difficulty in explaining interindividual variability in treatment response, illness course, and differential risk of adverse outcomes (Jin et al., 2025). These limitations are particularly evident in treatment-resistant depression (TRD), is defined as a failure to achieve an adequate, or clinically meaningful, response to an adequate trial of antidepressant therapy (Corral et al., 2025). The US Food and Drug Administration (FDA) and the European Medicines Agency (EMA) have adopted the definition to: inadequate response to a minimum of two antidepressants despite adequacy of the treatment trial and adherence to treatment (McIntyre et al., 2023).

Approximately 30%–55% of individuals with major depressive disorder meet criteria for TRD, though definitions vary across staging models, and no universally agreed-upon criteria exist. This patient subgroup exhibits greater chronicity, more pronounced functional impairment, and an increased risk of suicidal behavior, suggesting the presence of additional pathophysiological mechanisms not addressed by traditional therapeutic models. In this context, depression cannot be understood as a unitary entity but rather as a syndrome comprising multiple biological sub phenotypes modulated by metabolic, inflammatory, and environmental factors.

Suicide, in turn, cannot be conceptualized solely as an extreme consequence of depressive severity but rather as a complex, dynamic, and multifactorial behavioral phenomenon. While established risk factors such as prior attempts and hopelessness provide some predictive value, their limited precision at the individual level underscores the need to integrate objective biological dimensions that can identify states of persistent vulnerability (Franklin et al., 2017; Fazel and Runeson, 2020). Contemporary research models, such as the NIMH Research Domain Criteria (RDoC), propose understanding depression through transdiagnostic neurobiological domains. Among these domains, alterations in immune function and metabolic regulation have emerged as particularly promising areas of investigation. A growing body of evidence suggests that peripheral biological processes, rather than being mere epiphenomena, may actively contribute to the pathophysiology of depression through their effects on brain function (Woody and Gibb, 2015).

Within this framework, low-grade systemic inflammation and its interaction with lipid metabolism emerge as potentially important axes, particularly in treatment-resistant forms and in patients exhibiting metabolic comorbidities (Zorkina et al., 2024).

Lipid metabolism not only reflects the individual’s metabolic state but also acts as an active source of inflammatory signals capable of modulating brain function, neuroimmune, and behavior (Bernal‐Vega et al., 2023). This immunometabolic convergence offers an integrative framework for understanding the clinical heterogeneity of major depressive disorder and provides the rationale for exploring lipid metabolism as a key amplifier of inflammation relevant to the central nervous system.

The heterogeneity of major depressive disorder, its substantial suicide risk, and the limited predictive value of current diagnostic criteria underscore the need for objective biomarkers. Within this context, a lipid-based framework proposes that alterations in lipid metabolism, particularly involving ceramides, together with inflammatory and neurotrophic markers, contribute to neuroinflammation, treatment resistance, and suicidal vulnerability in MDD.

The present narrative review examines the evidence supporting ceramides and lipid metabolism as drivers of neuroinflammatory processes in major depression, integrates lipidomic and neuroinflammatory findings related to depressive pathology and suicidal behavior, addresses methodological challenges in the field, and outlines future directions toward precision psychiatry.

Among lipid mediators, ceramides deserve specific attention because they occupy a central position in sphingolipid metabolism and simultaneously regulate inflammatory amplification, membrane microdomain organization, blood-brain barrier integrity, mitochondrial stress, regulated cell death, and antidepressant-sensitive acid sphingomyelinase signaling. This combination of mechanistic centrality and emerging clinical detectability distinguishes ceramides from broader lipid classes and supports the specific focus of the present review.

## 2 Bibliographic search strategy

This manuscript was prepared as a narrative review. To identify relevant literature, we searched PubMed, Google Scholar, and ScienceDirect using combinations of the following terms: *ceramides*, *sphingolipid metabolism*, *neuroinflammation*, *major depressive disorder*, *treatment-resistant depression*, *suicidal behavior*, *kynurenine pathway*, and *mitochondrial dysfunction*. Priority was given to human studies, systematic reviews, meta-analyses, and recent translational work relevant to the conceptual framework of the review. Earlier foundational references were included when necessary to support key biochemical, neuroimmune, and neuroendocrine concepts. Because this is a narrative review, study selection was guided by conceptual relevance and interpretive value rather than by a formal systematic-review workflow.

## 3 Background and pathophysiological framework

### 3.1 Clinical burden of MDD and TRD

Depressive disorders encompass, according to the World Health Organization (WHO), a group of conditions characterized by persistently depressed mood or a marked loss of interest or pleasure in activities, often accompanied by somatic and cognitive changes that significantly impair functioning. In the Diagnostic and Statistical Manual of Mental Disorders (DSM-5), these disorders are classified separately from bipolar disorder (BPD) and are distinguished primarily by symptom profile, duration, and clinical course. Although the DSM-5 includes other subtypes, such as Disruptive Mood Dysregulation Disorder, Persistent Depressive Disorder, Premenstrual Dysphoric Disorder, and depressive conditions induced by substances or medical conditions, as well as unspecified depressive disorder, Major Depressive Disorder (MDD) remains the principal diagnostic category.

MDD is defined as the presence of severe depressive symptoms causing clinically significant distress or functional impairment. Diagnosis requires five of nine symptoms, occurring nearly every day during the same 2-week period, with at least one of the symptoms being depressed mood or anhedonia. In individuals with MDD, suicide risk may be present throughout depressive episodes. The most consistent risk factor is a history of suicide attempts or threats, although most completed suicides occur without prior attempts. Additional factors associated with increased risk include male sex, social isolation, living alone, and intense feelings of hopelessness (American Psychiatric Association, 2013). From an alternative classificatory perspective, the ICD-10 conceptualizes depressive disorder (F32–F33) primarily in terms of episode severity and longitudinal course, distinguishing levels of severity and defining recurrence based on periods of remission, with additional specification of remission status according to clinical evolution (World Health Organization, 2004).

Current diagnostic frameworks such as DSM-5 and ICD-10 remain clinically useful for identifying depressive disorders and guiding treatment decisions. However, because these classifications are primarily symptom-based, they have limited ability to capture the underlying biological heterogeneity of MDD and TRD. In the context of the present review, these diagnostic systems are therefore referenced mainly as clinical anchors, while the primary focus is placed on emerging immunometabolic and lipid-related mechanisms that may help explain disease persistence, treatment resistance, and suicidal vulnerability. For completeness, the formal DSM-5 diagnostic criteria and the ICD-10 classification framework are provided in Supplementary Tables S1, S2.

While both DSM-5 and ICD-10 provide standardized clinical frameworks, research-oriented models have attempted to integrate biological dimensions into the understanding of depression. The Research Domain Criteria (RDoC) framework proposed by the National Institute of Mental Health conceptualizes depression largely within the Loss construct of the negative valence system, emphasizing neurobiological and behavioral alterations that span multiple levels of analysis. Under this model, MDD involves disruptions in cortico-limbic circuits characterized by limbic hyperreactivity, reduced prefrontal control, and increased default mode network activity. These circuit-level alterations interact with disturbances in monoaminergic neurotransmission, hypothalamic-pituitary-adrenal (HPA) axis function, neuroimmune signaling, and autonomic function. Importantly, the RDoC framework highlights the heterogeneity of depressive disorders and supports the investigation of biologically informed subphenotypes, including those related to inflammatory and metabolic processes (Woody and Gibb, 2015).

TRD, however, presents conceptual challenges that extend beyond any single operational definition. The concept largely emerged from clinical trials such as the Sequenced Treatment Alternatives to Relieve Depression (STAR*D) study, which demonstrated that remission rates decline progressively with each successive treatment failure, suggesting a continuum of resistance rather than a discrete category. Several staging models have been proposed to operationalize this continuum, including Thase-Rush and Maudsley models, which stratify severity according to the number and adequacy of failed treatments. However, these models diverge considerably with respect to thresholds, episode duration, and severity criteria. Moreover, current definitions remain largely pharmacotherapy-focused and often overlook psychotherapy outcomes, patient-reported measures, and biopsychosocial factors such as childhood trauma or medical comorbidities that may critically influence treatment trajectories (McIntyre et al., 2023). This definitional heterogeneity complicates prevalence estimates, clinical trial design, and the identification of biologically meaningful subgroups.

Current regulatory guidance for antidepressant development reflects these limitations. The U.S. Food and Drug Administration (FDA) primarily relies on symptom-based endpoints measured through standardized rating scales such as the Hamilton Depression Rating Scale (HAM-D) or the Montgomery-Åsberg Depression Rating Scale (MADRS), typically across short-term trials lasting six to 8 weeks and longer maintenance phases of 6 months or more (Carmody et al., 2006). For TRD specifically, regulatory framework distinguishes between adjunctive therapy in partial responders and monotherapy in patients who have failed at least two prior antidepressant trials. Notably, no biomarkers are currently approved for patient stratification or treatment selection, clinical trial designs remain based almost exclusively on symptom ratings. This gap highlights the urgent need for validated biological markers capable of improving patient stratification and outcome prediction.

The absence of biologically informed classification contributes to several major obstacles in depression research and treatment development. Definitional heterogeneity complicates accurate prevalence estimates, limits comparability across clinical trials, and hinders the identification of predictive risk markers. It may also contribute to widespread but often unvalidated polypharmacy and to difficulties in distinguishing true treatment resistance from partial response. Consequently, many therapeutic development programs continue to focus on non-resistant populations, while multidimensional outcomes—such as quality of life, functional recovery, and sustained remission—remain insufficiently integrated into clinical evaluation frameworks (McIntyre et al., 2023).

MDD is also associated with substantial risk of suicidal behavior. Lifetime suicide risk among individuals with major depressive disorder has been estimated at approximately 15% (Orsolini et al., 2020) Importantly, many suicides occur without prior attempts, which limits the predictive utility of clinical history alone. Suicide is increasingly conceptualized not merely as an expression of depressive severity but as a complex behavioral outcome arising from interactions between biological vulnerability, emotional dysregulation, and environmental stressors. Traditional risk assessment distinguishes between static risk factors (such as prior attempts, family history, or demographic variables) and dynamic risk factors (such as acute stressors, hopelessness, or access to lethal means), yet even comprehensive models demonstrate limited predictive accuracy at the individual level. While serotonergic dysfunction has long been considered central to the neurobiology of suicide (Mann, 2013), growing evidence implicates additional systems, including inflammatory signaling and metabolic dysregulation, as contributors to suicide risk that may operate partially independently of mood state (Baldini et al., 2025).

Meta-analytic evidence supports the association between MDD and elevated suicide risk compared with non-depressed populations. A pooled analysis of 15 studies involving 85,768 individuals found significantly increased odds of suicidal ideation across multiple timeframes (monthly OR = 49.88; annual OR = 13.97; 2-week OR = 24.81; all p < 0.001), as well as elevated rates of suicidal planning (lifetime OR = 9.51, p < 0.001) and suicide attempts (lifetime OR = 3.45, p = 0.002). Interestingly, completed suicide did not differ significantly between groups (OR = 0.69, p = 0.50), which may reflect methodological challenges in capturing this outcome or indicate that factors beyond diagnosis influence lethality (Cai et al., 2021).

Within MDD populations, the severity of suicidality varies considerably. In a cohort of 1,410 patients examined by the European Group for the Study of Resistant Depression (GSRD), nearly half (46.67%) exhibited some degree of suicidality as measured by item 3 of the HAM-D scale. Severe suicidality (8.23% of patients) was associated with inpatient treatment, use of antipsychotics and benzodiazepines, melancholic features, and somatic comorbidities, whereas mild-to-moderate suicidality was linked primarily to greater depressive severity and treatment resistance (Dold et al., 2018). These findings underscore the marked heterogeneity of suicide risk within MDD populations and highlight the need for biologically informed stratification strategies capable of identifying high-risk subgroups.

### 3.2 Limitations of classical models and the need for translational biomarkers

The transition from suicidal ideation to attempt and completed suicide remains poorly predicted at the individual level. This limitation, combined with the clinical heterogeneity of MDD and the frequent absence of prior attempts, underscores the insufficiency of clinical judgment alone for risk stratification (Franklin et al., 2017). Objective biomarkers could complement clinical assessment by enabling earlier detection and more precise risk stratification of suicidal vulnerability in major depressive disorder (MDD). A neuroimaging study compared patients with MDD with suicidal ideation (MDDSI, n = 48) and without suicidal ideation (MDDNSI, n = 44), finding no differences in demographic or clinical variables. Resting-state functional magnetic resonance imaging with voxel-mirrored homotopic connectivity (VMHC) analysis showed increased VMHC in the bilateral superior frontal gyrus, putamen, inferior temporal gyrus, and cerebellum in the MDDSI group. VMHC in the inferior temporal gyrus and putamen correlated positively with suicidal ideation severity. Support vector machine classification using VMHC in the inferior temporal gyrus achieved 81.6% accuracy, increasing to 86.8% when all regions were combined. These results support VMHC as a potential biomarker of suicidal ideation, although replication in larger samples is required (Wang et al., 2025). In addition to neuroimaging markers, peripheral blood markers have emerged as potential indicators of suicide risk. Low serum BDNF differentiates non-suicidal MDD from MDD with suicidal ideation (SI) and correlates with SI severity (Khan et al., 2019). Elevated CRP, inflammatory markers, distinguishes suicidal from non-suicidal MDD and healthy controls (Miola et al., 2021), while High-sensitivity CRP (HsCRP) and ESR (Erythrocyte sedimentation rate) are elevated in suicide ideation and correlate with severity (Chang et al., 2017). TNF-α has been shown to predict SI at 12 weeks. A combined inflammatory/lipid panel (AAT, TRSF, HDL-c, APOA1) differentiates SI from non-suicidal MDD. Additionally, serotonergic alterations, including low CSF 5-HIAA, elevated kynurenine/tryptophan ratio, and reduced tryptophan, have been associated with suicide attempts (Johnston et al., 2022).

### 3.3 Neuro-immune-metabolism as mechanistic axes

MDD involves dysregulated communication between peripheral immune activity and central nervous system function. In a subset of patients, persistent low-grade systemic inflammation, reflected by elevations in circulating cytokines and acute-phase proteins, constitutes a chronic biological stressor capable of influencing brain function through neural, humoral, and barrier-related mechanisms. Within the central nervous system, microglia act as a key interface between immune signaling and neural plasticity. Current evidence indicates that microglial responses in depression cannot be adequately described by a binary polarization framework, but rather by a spectrum of activation states with distinct metabolic and functional profiles (Kang et al., 2020; García-Juárez et al., 2025). Importantly, immune activation in depression is increasingly recognized as tightly coupled to changes in endocrine and lipid metabolism. Neuroendocrine regulation has been associated with HPA axis dysfunction is also well-documented, with elevated cortisol in plasma and cerebrospinal fluid and abnormal dexamethasone suppression test responses observed in depression (Pandey and Dwivedi, 2012).

In the other hand, Lipid-derived mediators, particularly sphingolipids such as ceramides, have emerged as biologically plausible links between peripheral inflammation, microglial reactivity, and depressive symptomatology. In a cohort of 1,718 medication-naive patients with first-episode MDD, 20.14% had a history of suicide attempts. Compared with non-attempters, suicide attempters exhibited a proatherogenic lipid profile, characterized by higher total cholesterol, LDL-c, and triglycerides, and lower HDL-c, together with greater depressive and anxiety severity. After adjustment for demographic and clinical variables, elevated total cholesterol, reduced HDL-c, and higher HAMD and HAMA scores remained independently associated with suicide attempts. These findings indicate that proatherogenic lipid profiles, in combination with symptom severity, may represent a biological risk marker for suicidal behavior in early MDD (Ma et al., 2020). Taken together, these findings suggest that specific alterations in lipid metabolism, including classical plasma lipids and ceramides, could serve as biomarkers and potential therapeutic targets in major depression. Prior systematic review evidence indicates that certain ceramide species, particularly C18:0 and C20:0, are consistently associated with depressive symptoms (Dinoff et al., 2017).

### 3.4 Systemic inflammation and neuroinflammation in MDD/TRD

Elevated circulating proinflammatory cytokines have been associated with MDD, though findings are inconsistent across studies. A meta-analysis in adolescents with MDD found significantly elevated TNF-α compared with healthy controls (n = 222, Hedge’s g = 0.51, p < 0.01), while other cytokines including IL-1β, IL-6, and IFN-γ showed no significant differences (Jadhav et al., 2025). Notably, not all elevated cytokines in MDD are proinflammatory. A separate study measuring 18 serum cytokines in adults during major depressive episodes (57.1% discovery cohort n = 264; 70% replication cohort n = 93) found elevations in IL-10 and IL-27, both anti-inflammatory, as well as IL-15, which has mixed homeostatic and proinflammatory functions (Martinuzzi et al., 2021).

Meta-analyses in adult MDD populations provide the strongest available evidence for cytokine dysregulation in this disorder. Across 24 studies, significantly higher concentrations of TNF-α (WMD 3.97 pg/mL, 95% CI 2.24–5.71; p < 0.00001) and IL-6 (WMD 1.78 pg/mL, 95% CI 1.23–2.33; p < 0.00001) have been reported in depressed subjects compared with controls, while no significant differences were found for IL-1β, IL-2, IL-4, IL-8, IL-10, or IFN-γ (Dowlati et al., 2010). A cumulative meta-analysis of 58 studies confirmed elevated IL-6 (d = 0.54, p < 0.0001) and CRP (d = 0.47, p < 0.0001) in MDD with moderate heterogeneity; TNF-α showed a significant but unstable association (d = 0.40, p = 0.002) due to extensive between-study variability, and IL-1β showed no consistent association (d = −0.05, p = 0.86) (Haapakoski et al., 2015). Regarding treatment effects, antidepressant treatment has been associated with significant reductions in IL-6 (Hedges g = −0.454, p < 0.001), TNF-α (g = −0.202, p = 0.015), IL-10 (g = −0.566, p = 0.012), and CCL-2 (g = −1.502, p = 0.006) across 45 studies (N = 1,517), though these reductions were not consistently linked to treatment response (Köhler et al., 2017).

This heterogeneity is further illustrated by a primary study in 113 adult MDD patients, which found significant elevations in TNF-α (13.35 ± 1.24 vs. 12.90 ± 0.23 pg/mL; p < 0.001) and IL-6 (2.11 vs. 0.42 pg/mL; p < 0.001), alongside reduced HMGB1 (118.0 ± 28.1 vs. 130.2 ± 26.4 pg/mL; p = 0.018); both markers remained elevated after 6 weeks of antidepressant treatment without significant normalization, consistent with the heterogeneity observed at the meta-analytic level (Min et al., 2023).

That reflects differences in patient populations, illness stage, and measurement approaches, and underscores the need for standardized assessment frameworks. A similar pattern of inconsistency emerges in studies of central neuroinflammation.

### 3.5 Microglial states and neuroinflammatory heterogeneity in major depressive disorder

The role of microglia in MDD pathophysiology remains contested, with human studies yielding heterogeneous and sometimes contradictory findings. Rather than consistent inflammatory activation, the emerging picture suggests context-dependent microglial states that may differ between depression subtypes and, critically, between suicidal and non-suicidal presentations. Several lines of evidence suggest that microglia in MDD exhibit downregulation rather than classical activation. In cortical gray matter, 92 microglial genes are differentially expressed, predominantly downregulated (81 genes), including CD163, MKI67, SPP1, CD14, FCGR1A/C, and complement components C1QA/B/C. Also, attenuation of microglial effector pathways, including complement-mediated phagocytosis (reduced CD163), and neuronal inhibitory signals, as CD200 mRNA is significantly increased (p = 0.0009) and CD47 protein is elevated in synaptic fractions (p = 0.0396), with a trend toward increased CD47 mRNA (p = 0.068), suggesting active suppression of microglial inflammatory responses in cortical gray matter (Scheepstra et al., 2023) Similarly, microglia isolated from multiple brain regions (subventricular zone, thalamus, temporal and frontal lobes) in post-mortem MDD tissue retained regional heterogeneity without global separation between cases and controls. However, MDD microglia showed increased homeostatic markers (P2Y12, TMEM119) and decreased activation markers (HLA-DR, CD68), particularly in the subventricular zone. These findings suggest that in MDD, microglia adopt a more homeostatic or reparative state without clear proinflammatory cytokine elevation (Böttcher et al., 2020).

The largest transcriptomic study to date reinforces this pattern. Using RNA sequencing and cellular deconvolution in 458 subjects (242 with recurrent MDD and 216 controls), slight reductions (≤1%) in estimated microglial proportions were observed in the sub genual anterior cingulate cortex and amygdala. No robust microglial transcriptomic signatures or evidence of marked inflammatory activation were identified. Together, these findings suggest that microglial changes are subtle and more likely reflect adaptations or downstream consequences rather than primary drivers of MDD pathophysiology (Goes et al., 2025) Contrasting evidence, however, supports pathogenic microglial involvement through epigenetic mechanisms such Circular RNAs (circRNAs), a class of RNAs generated from protein coding genes by back-splicing, playing crucial roles in various pathological processes (Zhu et al., 2021) The circRNA circ-UBE2K is elevated in both MDD patients and murine models, promoting dysfunctional microglial activation and neuroinflammation. Functionally, its overexpression aggravates depressive behaviors while its silencing attenuates them. Circ-UBE2K forms a complex with HNRNPU, increasing UBE2K expression and favoring depression progression (Cai Y. et al., 2024). Similarly, the epigenetic repressor PCGF1 is downregulated specifically in microglia following chronic unpredictable mild stress (CUMS) in mice, associating with microglial activation, increased proinflammatory cytokines (IL-1β, IL-6, TNF-α), neuronal damage in CA1, and depressive behaviors. Mechanistically, PCGF1 represses MMP10 transcription through histone modifications H2AK119ub and H3K27me3 via PRC1/PRC2, thereby inhibiting NF-κB/MAPK pathways. Microglial-specific PCGF1 overexpression reverses both neuroinflammation and depressive phenotypes. Notably, plasma PCGF1 levels correlate negatively with clinical severity in adolescents with MDD, supporting translational relevance (Li et al., 2025). It is important to consider in the etiology of MDD that environmental factors may indirectly modulate behavior, as microglia can respond to systemic stimuli. Also, peripheral inflammation and stress exacerbate microglia-mediated neuroinflammation and impairs synaptic plasticity (Robledo-Montaña et al., 2024).

In addition to morphological alterations, environmental epigenetic effects, synaptic modulation, and central and peripheral activation states, neuroinflammation is accompanied by dynamic shifts in cellular and functional phenotypes differentially impact protein accumulation, plasticity deficiency, and synaptic pruning (Fujikawa and Tsuda, 2023) The apparent contradiction between microglial downregulation and activation may reflect phenotypic heterogeneity within MDD itself. A critical recent study clarifies this by distinguishing suicidal from non-suicidal presentations. Using post-mortem hippocampal samples from controls (C), MDD patients without suicide (D-S), and MDD patients who died by suicide (D + S), cell-type-specific transcriptomic analysis using RNA-seq and snRNA-seq-based deconvolution revealed that marked immuno-inflammatory activation was present only in the suicidal phenotype. In pyramidal neurons, suicide-specific genes enriched interleukin signaling, inflammatory response, and immune defense pathways, with hubs including TLR2, CD86, CXCL8, and ITGAM. Microglia and endothelium similarly showed enrichment in IL-10 and IFN-γ signaling. Crucially, these inflammatory signatures were absent in non-suicidal MDD (Francis et al., 2025).

These findings suggest that neuroinflammation may not be a core feature of MDD *per se*, but rather a marker of suicidal vulnerability within depressed populations. This distinction has important implications for biomarker development and therapeutic targeting: inflammatory signatures may be more relevant for suicide risk stratification than for depression diagnosis broadly. It also underscores the importance of phenotypic precision in MDD research, as pooling suicidal and non-suicidal patients may obscure biologically meaningful differences.

### 3.6 Stress-related HPA axis activity and associated immunometabolic changes

Chronic stress represents a central pathway linking HPA axis dysregulation to both inflammation and lipid metabolism alterations in depression. Sustained HPA activation elevates cortisol, which in turn promotes systemic and neuroinflammation through release of proinflammatory cytokines (IL-1β, IL-6, IL-18) and activation of NF-κB and MAPK pathways. These processes affect neuroplasticity, reduce BDNF, compromise blood-brain barrier integrity, and damage mood-regulating circuits (Liu et al., 2025).

Importantly, chronic cortisol excess also induces profound lipid metabolism alterations. Metabolomic analysis in Cushing syndrome, an extreme model of sustained hypercortisolemia, identified 93 significantly altered metabolites, including increased triacylglycerols, ceramides, glycerophospholipids, and cholesterol esters. Urinary cortisol excretion independently predicted concentrations of multiple lipids and amino acids, with disruptions observed in the Kennedy pathway and Lands cycle, key routes for membrane phospholipid synthesis and remodeling (Vega-Beyhart et al., 2021).

These findings suggest that HPA axis dysfunction may contribute to the lipid alterations observed in MDD, providing a mechanistic link between stress, inflammation, and the immunometabolic disturbances central to this review.

In TRD, dysregulation of the HPA axis is commonly observed and is characterized by prolonged glucocorticoid resistance, hypercortisolemia, and elevated proinflammatory cytokine levels. These alterations contribute to neurotoxic effects, hippocampal atrophy, and impaired neuronal plasticity (Bose et al., 2025) In recurrent MDD, increased evening cortisol levels have been associated with a reduced KYN/TRP ratio, suggesting that HPA axis dysfunction preferentially influences tryptophan metabolism during recurrent depressive episodes (Sorgdrager et al., 2017). Conversely, patients with MDD have been reported to exhibit lower levels of kynurenine and quinolinic acid, alongside elevated kynurenic acid levels and increased KYNA/QUIN ratios. These alterations were associated with cognitive impairments, particularly in working memory performance (Pan et al., 2025) Together, this evidence illustrates how chronic stress and HPA axis dysfunction interact with immunometabolic pathways and the TKP, thereby modulating depressive vulnerability and contributing to treatment resistance.

In the context of suicidal ideation, in a systematic review the relationship between HPA axis, cortisol and suicidality is evaluated in studies from 1980 to 2020, where 36 studies in adults and adolescents with MDD and suicidal behavior were analyzed. Through DST, TSST, PCR and post-mortem studies in prefrontal cortex, hippocampus and amygdala, it was documented that non suppression in the DST multiplies suicide risk 14 times. Chronically elevated cortisol may contribute to NF-κB-related inflammatory signaling and, indirectly, to pathways linked to acid sphingomyelinase activation and increased neuroactive ceramides. Post-mortem glucocorticoid resistance decreases GR expression in PFC, linking HPA dysfunction with ceramide mediated neurotoxicity and suicidability (Berardelli et al., 2020). It has been reported that HPA axis dysfunction is associated with suicidal severity in 568 hospitalized Caucasian patients with MDD (51.8% women, mean 47.2 ± 13.4 years) with acute depressive episode, stratified in non-suicidal (NS, n = 143), weary of life (WoL, n = 171), suicidal ideators (SI, n = 192) and suicide attempt (SA, n = 62). Through combined dex CRH test in venous blood, the WoL group showed cortisol (CAUC) and ACTH (AAUC) significantly higher against NS (p = 0.043 and p = 0.035 respectively). A higher number of previous suicide attempts has been reported to correlate negatively with cortisol response (CAUC rs = −0.141, p = 0.035) (Hennings et al., 2021). Although this observation does not directly implicate sphingolipid metabolism, it may be compatible with broader stress-related metabolic alterations. Experimental evidence suggests that acute hypercortisolemia can promote NF-κB–related inflammatory signaling and may indirectly contribute to acid sphingomyelinase (aSMase) activation and increased production of neuroactive ceramides (Demarchi et al., 2005).

Additional evidence comes from studies evaluating offspring of parents with mood disorders. In a cohort of 208 young individuals (mean age 23.3 ± 5.3 years) from 134 probands with mood disorders, participants were stratified into suicide attempt (SA, n = 20), suicide-related behavior (SRB, n = 20), and non-suicidal groups (NS, n = 168), and compared with 35 healthy controls. Using the Trier Social Stress Test (TSST) with salivary cortisol measured by radioimmunoassay, the SA group showed significantly reduced total cortisol output (AUCG) compared with controls (β = −0.47, p = 0.01, ES = −0.75), as well as lower basal cortisol levels (β = −0.45, p = 0.002, ES = −0.87). This hypocortisolaemia pattern in individuals with a history of suicide attempts may reflect chronic HPA-axis dysregulation or exhaustion. While such alterations may be consistent with persistent inflammatory activation, their relationship with sphingolipid metabolism and ceramide signaling remains indirect and has not yet been demonstrated in human studies (Melhem et al., 2016).

So far no direct relationship was found, this study in animal models shows something that can associate the HPA axis, depression, ceramides, a topic that is also developed in the text, which is mitochondrial function, this with the microbiota. In this study where corticosterone, microbiota, ceramides and hippocampal mitochondrial dysfunction are evaluated in a murine depression model in C57BL/6J mice chronically treated with corticosterone (CORT, 36 days) showed depressive behavior, intestinal dysbiosis with *Lactobacillus* (20.42% increases to 31.56%, p = 0.027) and Bifidobacterium (1.20% increases to 3.02%, p = 0.035), and increased fecal ceramides. Hippocampal RNA seq identified 16 of 17 OXPHOS genes decreased (p < 0.05), with decreased mitochondrial potential and ATP. Inhibition of ceramide synthesis with myriocin reversed depressive behavior and restored mitochondrial function, establishing the direct mechanistic chain where hypercortisolemia generates dysbiosis, increases intestinal ceramides, impairs hippocampal OXPHOS and produces depression, linking HPA dysfunction with ceramide metabolism and mitochondrial energetics (Wang et al., 2024).

Overall, available evidence supports a biologically plausible connection between HPA-axis dysregulation, inflammatory activation, and ceramide metabolism. However, in the context of suicidality, these links remain largely indirect and inferential. Direct human studies simultaneously measuring cortisol dynamics, ceramide species, and suicidal phenotypes are still lacking. Therefore, the HPA-ceramide-suicidality axis should currently be viewed as a promising mechanistic hypothesis rather than an established biomarker pathway.

### 3.7 Tryptophan/kynurenine pathway and ceramides

The tryptophan/kynurenine pathway (TKP) is the main route of tryptophan metabolism, regulating key biological processes. Tryptophan is constitutively oxidized in the liver by tryptophan-2,3-dioxygenase (TDO), while inducible Indoleamine 2,3-dioxygenase (IDO) in other cells increases formation of kynurenine metabolites under pathological conditions, including inflammation (Badawy, 2017).

At the transcriptional level, IDO1 transcription is strongly upregulated by interferon-γ and synergistically enhanced by TNF-α through NF-κB-dependent mechanisms (Robinson et al., 2006). Ceramide generated via sphingomyelinase activation also promotes NF-κB signaling and inflammatory cytokine production (Schütze et al., 1992), providing a mechanistic framework by which ceramide-driven inflammation could amplify IDO1 activation and contribute to dysregulation of the tryptophan-kynurenine pathway (TKP) in chronic inflammatory conditions such as major depressive disorder. Several enzymes within the kynurenine pathway, including kynureninase and kynurenine aminotransferases, require pyridoxal 5′-phosphate (PLP) as a cofactor (Badawy, 2017). Crucially, PLP is also required for key enzymes involved in sphingolipid metabolism, such as serine palmitoyl transferase and sphingosine-1-phosphate lyase (Bourquin et al., 2011; Lowther et al., 2012) This shared cofactor dependence represents a highly probable biochemical point of convergence between the kynurenine and sphingolipid pathways. Under inflammatory conditions where PLP availability is frequently reduced (Sakakeeny et al., 2012; Chiang et al., 2005), this metabolic bottleneck could drive the concurrent dysregulation of both lipid metabolism and the kynurenine pathway, providing a unified mechanism for the metabolic shifts observed in depressive states. Clinical evidence supports this immunometabolic link across depressive phenotypes. A systematic review and meta-analysis of immune activation studies in adult patients with chronic medical illnesses receiving interferon-α treatment reported decreased tryptophan levels and increased KYN over time, changes that paralleled increases in depressive symptom scores (Hunt et al., 2020). Furthermore, a recent meta-analysis further demonstrated that current MDD is characterized by reduced levels of tryptophan, kynurenine, kynurenic acid (KYNA), and their ratios relative to quinolinic acid (QA) and 3-hydroxykynurenine (3-HK), alongside an increased KYN/TRP ratio, particularly in unmedicated patients. In this context, KYNA showed a negative correlation with depressive severity and increased following treatment, whereas QA levels remained stable (Ou et al., 2023).

Collectively, these findings indicate that alterations in the TKP are closely linked to inflammatory processes and may interact with lipid metabolism to modulate neuropsychiatric risk. As such, components of this pathway may serve as functional biomarkers of immunometabolic dysfunction in mood disorders and suicidality.

This inflammatory diversion of tryptophan metabolism is particularly pronounced in severe clinical phenotypes associated with high suicide risk, such as adolescent and postpartum depression. In adolescents with MDD and high suicide risk, the KYN/TRP ratio, reflecting IDO activity, has been shown to correlate with circulating levels of IL-1β, IL-6, IL-10, and TNF-α. In parallel, the 3-HK/KYN ratio, indicative of kynurenine 3-monooxygenase (KMO) activity, has been associated with IL-8, suggesting TKP dysregulation linked to inflammation and suicidality (Yang et al., 2025). Similarly, in a cohort study of women hospitalized for severe post-partum depression, a condition that differs etiologically from major depressive disorder but shares clinical features and suicide risk, inflammatory activation was associated with dysregulation of the kynurenine pathway and reduced peripheral serotonin levels. Increased concentrations of pro inflammatory cytokines including IL 6 and IL 8 were linked to higher odds of depression, while tryptophan metabolism was preferentially diverted toward the kynurenine pathway rather than serotonin synthesis. Notably, reduced serotonin levels were independently associated with suicidal behavior, suggesting that inflammation driven alterations in tryptophan metabolism may contribute to suicide risk through immunometabolic mechanisms (Achtyes et al., 2020).

Additionally, the downstream consequences of TKP dysregulation converge with ceramide-mediated effects at the synaptic level. Quinolinic acid, a TKP metabolite, acts as an NMDA receptor agonist promoting excitotoxicity (Hestad et al., 2022), while ceramides act concurrently to modulate NMDA receptor trafficking and synaptic insertion through nSMase2 activation (Wheeler et al., 2009). Both pathways also contribute to mitochondrial oxidative stress and impaired bioenergetics, further amplifying neuronal vulnerability in depression (Jamil and Cowart, 2023; Iwata, 2019; Lugo-Huitrón et al., 2013).

Collectively, these findings indicate that alterations in the TKP could be linked to inflammatory processes *and* ceramide metabolism through shared inflammatory mediators, cofactor dependence, and convergent effects on glutamatergic signaling and mitochondrial function. As such, components of both pathways may serve as complementary functional biomarkers of immunometabolic dysfunction in mood disorders and suicidality.

### 3.8 Inflammation-mitochondria-bioenergetics

Mitochondrial dysfunction represents a central pathophysiological feature of MDD and bipolar disorders, with direct consequences for cellular energy production and increased oxidative stress (Jia et al., 2024; Giménez-Palomo et al., 2021). Therapeutic agents that enhance mitochondrial function or strengthen antioxidant defenses may therefore have value as adjunctive treatments, particularly in patients with treatment-resistant forms of illness.

Persistent inflammatory activity can further aggravate mitochondrial impairment by activating indoleamine 2,3-dioxygenase and increasing the production of neurotoxic kynurenine metabolites. This process compromises neuronal energy efficiency and may contribute to cognitive dysfunction and the persistence of depressive symptoms.

### 3.9 Interconnected pathways in MDD and TRD linking inflammation, tryptophan metabolism, and ceramide signaling, mitochondrial dysfunction

The tryptophan-kynurenine pathway, HPA axis dysregulation, inflammation, and mitochondrial dysfunction converge in MDD and TRD, jointly modulating depressive and cognitive vulnerability. Inflammatory activation of indoleamine 2,3-dioxygenase increases the production of neurotoxic kynurenine metabolites, with downstream effects on serotonergic availability and excitotoxic mechanisms mediated by N-methyl-D-aspartate receptors (Ramírez et al., 2018).

Concurrently, HPA axis dysfunction and sustained cortisol elevation are associated with reductions in the KYN/TRP ratio and alterations in neuronal plasticity (Sorgdrager et al., 2017). Mitochondrial impairment further contributes by disrupting energy production and increasing oxidative stress (Jia et al., 2024; Giménez-Palomo et al., 2021).

Ceramide metabolism intersects with each of these axes. At the molecular level, ceramides amplify NF-κB-dependent cytokine signaling that drives IDO activation, share cofactor dependence on PLP with key TKP enzymes, and converge with quinolinic acid on NMDA receptor-mediated excitotoxicity and mitochondrial oxidative stress (Badawy, 2017; Hestad et al., 2022; Wheeler et al., 2009). Furthermore, sustained hypercortisolemia, as observed in MDD and particularly in TRD, induces ceramide accumulation alongside broader lipid metabolic disruption (Vega-Beyhart et al., 2021). These observations position ceramides as a mechanistic node linking HPA axis dysfunction, inflammatory activation of the TKP, and bioenergetic impairment within a unified immunometabolic framework.

Taken together, these interconnected systems outline a functional framework in which inflammation, altered tryptophan metabolism, ceramide-mediated signaling, bioenergetic dysfunction, and cognitive disturbances interact. This integrative perspective provides a basis for the development of personalized therapeutic strategies and supports future translational research efforts.

### 3.10 Lipid-associated immune signaling in experimental and neuroinflammatory models

Inflammatory processes have been linked to lipids themselves being recognized as damage-associated molecular patterns (DAMPs), directly linking lipid metabolism to innate immune activation. Lipids and lipoproteins act as direct modulators of immune responses promoting metabolic disorders (Ho et al., 2019) and neuroinflammation, where accumulation of free cholesterol in the promotes a neurotoxic astrocyte phenotype characterized by increased C3, greater reactivity, and morphological atrophy, linking neuroinflammation and cognitive impairment mediated by lipids (Niu et al., 2025). Also, saturated fatty acids, particularly palmitic acid, as well as free cholesterol, can activate pattern recognition receptors such as TLR4 and NLRP3 in the brain in various study models (Saipuljumri et al., 2025; Zhang et al., 2025; Li X. et al., 2024; Goldklang et al., 2012; Nicholas et al., 2017), producing behavioral changes as anxiety-like behavior in animal models and establishing lipids as active modulators of neuroinflammation (Moon et al., 2014). Also, Low density lipoproteins (LDL), particularly in its oxidized form (oxLDL), activates pattern recognition receptors including TLR4 and scavenger receptors on macrophages and microglia, promoting proinflammatory cytokine release (Stewart et al., 2010).

Thus, downregulation of the TLR4 pathway may represent a potential therapeutic strategy (Hu et al., 2022).

A key mechanism linking lipid accumulation to microglial dysfunction has been described in aging. In both mouse (3 vs. 20 months) and human post-mortem brains (22 vs. 67 years), hippocampal microglia accumulate lipid droplets enriched in glycerol lipids (TAG/DAG/MAG; 44.4%) and depleted in cholesterol esters (0.7%). These lipid droplet–accumulating microglia (LDAM) display a distinct transcriptomic profile (692 differentially expressed genes), with upregulation of phagosomal maturation, increased ROS/NO production, and enhanced fatty acid β-oxidation. Functionally, LDAM exhibit impaired phagocytosis, elevated oxidative stress, and increased secretion of proinflammatory cytokines (TNF-α, IL-1β, IL-6, CCL3) at baseline and after LPS stimulation. Notably, LPS administration reproduces LDAM formation, indicating that peripheral inflammatory signals can induce this dysfunctional microglial phenotype (Marschallinger et al., 2020).

These findings suggest a potential mechanistic relationship between lipid accumulation, innate immune activation, and microglial dysfunction that may be relevant to neuroinflammatory states including depression.

Lipid metabolism not only constitutes an energetic and structural axis but also acts as an active source and amplifier of systemic and central inflammation. In context of dyslipidemia or obesity, specific lipids acquire immunomodulatory properties and are recognized as endogenous damage signals (DAMPs) (Choi et al., 2025). Saturated fatty acids derived from energy-dense diets also modulate neuroinflammation through direct effects on microglia. Prenatal exposure to a cafeteria-type diet in animal models programmed increased microglial complexity and activation in the nucleus accumbens, associated with type I interferon signaling. *In vitro*, palmitic acid potentiated microglial inflammatory responses, increasing expression of Ifit1, IL-1β, and IL-6, as well as phagocytic capacity during early brain development (Montalvo-Martínez et al., 2022).

As outlined above, MDD and TRD are biologically heterogeneous conditions in which immune, metabolic, and stress-related alterations intersect with suicide risk. Importantly, these signatures are not intrinsic but are strongly modulated by clinical and lifestyle confounders, including BMI, diet, diabetes, and drug use, which directly shape lipid metabolism, immune activation, and HPA axis function and critically condition biomarker interpretation.

### 3.11 Adiposity functions as an active biological modifier in MDD

In MDD, obesity is associated with greater clinical severity. Higher BMI correlates with increased suicidality, earlier disorder onset, longer lifetime psychiatric hospitalizations, and greater medical comorbidity. Overweight and obese patients show a trend toward higher treatment resistance, indicating that excess adiposity may exacerbate illness burden and complicate therapeutic response (Kraus et al., 2023). This could be explained by secreting adipokines as proinflammatory cytokines (IL-6, TNF-α) produced for adipose tissue, and harboring immune cells including regulatory T cells (Elkins and Li, 2022). Excess adiposity promotes chronic low-grade inflammation associated with dyslipidemia, elevated triglycerides, small dense LDL, and increased ceramides (Rahimlou et al., 2016; Xia et al., 2014). Diets rich in saturated fats and ultra-processed foods elevate fatty acids such as palmitic acid, s mentioned above, activating TLR4 and NLRP3 thereby amplifying systemic and central inflammation (Saipuljumri et al., 2025; Dong et al., 2020).

This pharmacogenetic GWAS shows that second-generation antipsychotics induce lipid and BMI changes mediated by genetic vulnerability, identifying variants (ABCG2, APOA5, SLC2A9) linked to dyslipidaemia and weight gain. In 669 Chinese patients followed up to 18.7 years, multiple SGA-associated SNPs influenced LDL, HDL, and triglycerides. Although conducted in schizophrenia, these findings are highly relevant to MDD/TRD where SGAs are used as adjuncts, suggesting treatment-related adiposity and metabolic disruption may be genetically modulated and contribute to inflammation, HPA dysfunction, and clinical burden (Wong et al., 2025).

Also, Diabetes Mellitus Type II (DMII) profoundly alters lipid metabolism, favoring hypertriglyceridemia, increased atherogenic LDL, and systemic metabolic dysfunction that interacts with chronic inflammation. In 37,040 NHANES participants, diabetes prevalence was higher in depressed than non-depressed individuals (21.26% vs. 13.75%), and adjusted diabetes risk increased with depressive severity (OR 1.22 mild, 1.62 moderate, 1.52 severe). The comorbidity of diabetes and depression doubled total mortality (HR = 2.09) (Cai J. et al., 2024) Saturated fatty acids and lipopolysaccharides can activate toll-like receptor 4 (TLR4) in adipose tissue, triggering inflammatory pathways including nuclear factor-κB (NF-κB) and c-Jun N-terminal kinase (JNK), linking lipid accumulation to innate immune activation (Mello et al., 2023) In this context, adipose JNK1/2 activity has been inversely correlated with insulin sensitivity (M, r = −0.54, P < 0.05), independently predicting insulin resistance (P = 0.02), and highlighting its role as a key mediator connecting adiposity, metabolic dysfunction, and inflammatory signaling (Sourris et al., 2009).

**FIGURE 1:**
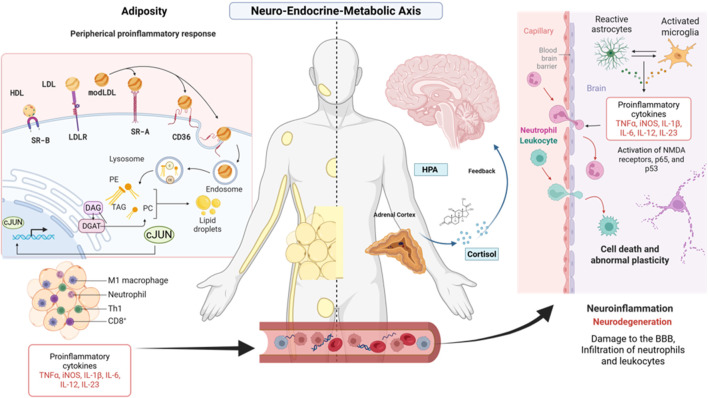
Ceramide metabolism and diversity. This figure illustrates the major ceramide biosynthetic pathways and CerS enzymatic specificity. The *de novo* pathway begins in the endoplasmic reticulum (ER) with the condensation of serine and palmitoyl-CoA. The sphingomyelinase (SMase) pathway generates ceramide through hydrolysis of sphingomyelin at the plasma membrane and lysosomes, while the salvage pathway recycles lysosomal sphingosine to produce new ceramide. Ceramide synthases (CerS1–CerS6) determine fatty acid chain length: CerS1, CerS5, and CerS6 produce long-chain ceramides (C16, C18) linked to pro-apoptotic signaling, whereas CerS2 generates very long-chain ceramides (C24) associated with membrane stability and homeostasis. High-density lipoprotein (HDL) can modulate ceramide levels by promoting ceramide efflux, linking lipid transport to systemic ceramide regulation. CerS (Ceramide Synthases), C16/C18/C24: ceramide carbon chain lengths (Palmitoyl, Stearoyl, and Lignoceric), SM (Sphingomyelin), aSMase (Acid Sphingomyelinase), nSMase (Neutral Sphingomyelinase), S1P (Sphingosine-1-Phosphate).

As illustrated in Figure 1, major depressive disorder and treatment-resistant depression involve complex interactions between systemic inflammation, HPA axis dysregulation, and lipid metabolism alterations. Brain microglia display heterogeneous states influenced by stress, adiposity, and environmental factors, while lipid mediators such as ceramides link peripheral inflammation to neuronal dysfunction, synaptic alterations, and suicide risk. These immunometabolic axes integrate central and peripheral mechanisms, providing a framework for identifying biomarkers and potential therapeutic targets in MDD and TRD.

Together, these confounders act as active biological determinants shaping inflammation, lipid metabolism, and central nervous system dysfunction in MDD/TRD. Current evidence converges on inflammatory lipid signalling as a core axis, ceramides potentially linking chronic stress, adiposity, inflammation, and suicidality, suggesting their possible role as mechanistic biomarkers worthy of further investigation.

## 4 Metabolism, signalling, and biogenesis of ceramides

Ceramides are the central building blocks of all sphingolipids, generated through three main pathways: *de novo* synthesis, the sphingomyelinase pathway, and the salvage pathway (Chinnappa et al., 2025) The *de novo* pathway, located primarily in the endoplasmic reticulum, begins with condensation of serine and palmitoyl-CoA by serine palmitoyl transferase (SPT) to form 3-ketosphinganine, which is reduced to sphingosine. Ceramide synthases (six isoenzymes with specificity for different acyl-CoAs) then acylate sphingosine to generate dihydroceramide, subsequently desaturated by dihydroceramide desaturase to produce ceramides (Mullen et al., 2012). Newly formed ceramides are transported to the Golgi apparatus via vesicles or ceramide transfer protein (CERT), where they are converted to sphingomyelin and glycosphingolipids before proceeding to the plasma membrane. The sphingomyelinase pathway generates ceramide from sphingomyelin hydrolysis in plasma membrane and lysosomes, while the salvage pathway recycles lysosomal sphingosine to the endoplasmic reticulum for new ceramide synthesis. Mammalian cells contain over 50 structural ceramide forms, differing primarily in acyl chain length and characteristics (Perera et al., 2016).

Long-chain ceramides such as C16 are abundant, naturally occurring forms integrated into cell membranes with specific physiological functions, whereas short-chain ceramides (C6, C10) are primarily used as experimental analogs. This structural diversity confers distinct functions depending on subcellular localization and cellular context (Wattenberg, 2018).

The functional significance of chain length is evident in clinical associations. Among plasma ceramides, C16:0 associates with adverse cardiovascular events, while very-long-chain species (C22:0, C24:0) and the C24:0/C16:0 ratio correlate with better cardiovascular health but also with cancer and neuroinflammation (de Wit et al., 2019; Mitchell et al., 2024; Zatloukal et al., 2024).

**FIGURE 2:**
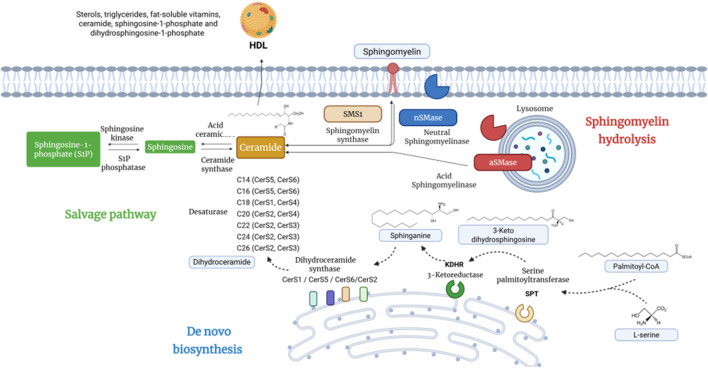
Neuro-immune-metabolism as mechanistic axes. This schematic depicts how chronic low-grade systemic inflammation, HPA axis dysregulation, and lipid metabolism alterations converge to influence central nervous system function in MDD and TRD. Peripheral proinflammatory cytokines, dyslipidemia, and saturated fatty acids activate microglia through TLR4/NLRP3 and NF-κB/MAPK/C-Jun pathways, impairing synaptic plasticity and promoting neuroinflammation. Lipid mediators, particularly ceramides, link adiposity, stress, and inflammation to depressive severity and suicidal vulnerability, while HDL provides protective effects by promoting ceramide efflux. Microglial responses span a spectrum from homeostatic to pathogenic, modulated by environmental, metabolic, and genetic factors, highlighting their central role in immunometabolic dysregulation.

The mechanisms of ceramide metabolism, including *de novo* synthesis, sphingomyelinase-mediated generation, and salvage pathways, are summarized in Figure 2. For clarity, key abbreviations are defined as follows: ER (Endoplasmic Reticulum), site of *de novo* synthesis; CerS (Ceramide Synthases), enzymes determining acyl chain length (CerS1–CerS6); C16/C18/C24, ceramide carbon chain lengths (Palmitoyl, Stearoyl, and Lignoceric, respectively); SM (Sphingomyelin), main ceramide reservoir in membranes; aSMase (Acid Sphingomyelinase), lysosomal/extracellular acid sphingomyelinase; nSMase (Neutral Sphingomyelinase), plasma membrane-associated neutral sphingomyelinase; and S1P (Sphingosine-1-Phosphate), lipid antagonistic to ceramide in the sphingolipid rheostat.

Lipidomic evidence from clinical human populations reinforces this rationale. A targeted lipidomic study in 67 patients with major depressive disorder or bipolar disorder and 405 healthy controls found significant elevations of multiple plasma ceramide species, C16, C18, C20, C22, and C24, in the patient group. Levels were associated with metabolic burden and antidepressant medication use, with no correlation with episode severity, suggesting a baseline disruption of sphingolipid metabolism in human affective disorders, independent of acute clinical state (Brunkhorst-Kanaan et al., 2019). Accumulated evidence on ceramides in mood disorders has been systematized in a systematic review summarizing clinical and preclinical findings that identify consistent alterations in specific species, particularly C18:0 and C20:0, in patients with major depression. As mentioned above, this suggest that human evidence remains limited and heterogeneous, whereas, the biological signal is sufficiently reproducible to position ceramides as priority candidates among lipid mediators relevant to translational psychiatry (Dinoff et al., 2017) Also, at the population level, a lipidomic study identified significant associations between plasma sphingolipid concentrations and depressive and anxiety symptoms, with specific ceramide species correlating with symptom burden in a community-based sample, extending the external validity of findings derived from selected clinical cohorts (Demirkan et al., 2013).

It should be noted, however, that direct evidence linking ceramides to suicidality in humans remains extremely limited. Available studies are exploratory and metabolomic in nature, with small sample sizes, no consistent replication, and no longitudinal designs. This qualitatively differentiates the state of evidence from that of classical serum lipids, cholesterol, HDL, LDL, triglycerides, which have robust meta-analyses in the context of suicide and cannot be directly extrapolated to the sphingolipid pathway.

### 4.1 Ceramides as central mediators of regulated cell death and stress responses

**FIGURE 3:**
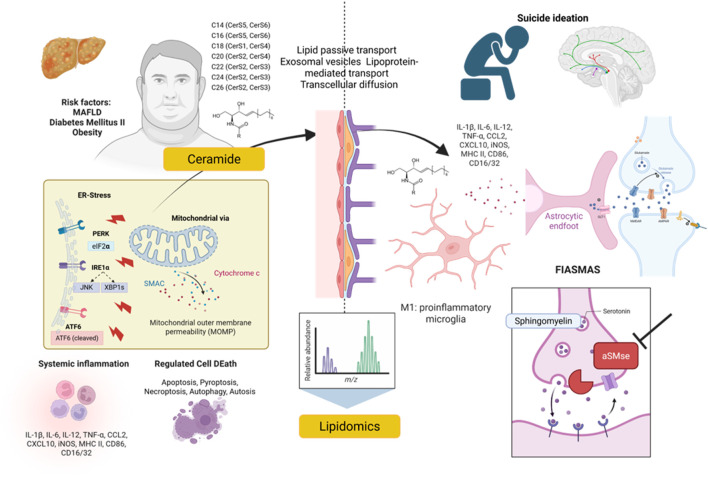
Ceramides as a regulator with mechanism involved in MDD. Ceramides orchestrate multiple forms of regulated cell death through caspase-dependent and caspase-independent mechanisms. They promote apoptosis via ROS generation, mitochondrial dysfunction, cytochrome c release, partial caspase activation, and AIF nuclear translocation, and induce inflammatory pyroptosis through NLRP3 inflammasome activation. Ceramides also trigger necroptosis via MLKL activation and drive autophagy-dependent cell death and lethal mitophagy through LC3 interactions. Beyond acute death pathways, ceramides sustain endoplasmic reticulum stress and mitochondrial dysfunction, linking lipid dysregulation to inflammation, neurodegeneration, and potential vulnerability in mood disorders. AMPA, $\alpha$-amino-3-hydroxy-5-methyl-4-isoxazolepropionic acid receptor; AIF, apoptosis-inducing factor; ASMase, acid sphingomyelinase; BBB, blood-brain barrier; BDNF, brain-derived neurotrophic factor; BMI, body mass index; CerS, ceramide synthase; Cyto C, cytochrome c; DAMPs, damage-associated molecular patterns; ER, endoplasmic reticulum; FIASMAs, functional inhibitors of acid sphingomyelinase.

Ceramides regulate multiple forms of caspase-dependent and caspase-independent mechanisms of regulated cell death (RCD) (Galluzzi et al., 2018) (see Figure 3) Ceramide-induced apoptosis has been characterized in human mesenchymal stem cells derived from adipose tissue (hASCs), where it occurs in a dose- and time-dependent manner through ROS generation, mitochondrial dysfunction, cytochrome c release, partial caspase activation, and nuclear translocation of AIF, combining caspase-dependent and independent pathways (Park et al., 2011).

In human endothelial cells (HUVECs), C8 ceramide induces pyroptosis through NLRP3 inflammasome activation. This process involves the ROS-TXNIP-NLRP3 pathway, leading to caspase-1 activation, GSDMD cleavage, and release of IL-1β and IL-18, ultimately increasing vascular permeability. Inhibition of NLRP3, TXNIP, or ROS reverses these effects, confirming a regulated inflammatory mechanism (Liu et al., 2022).

Ceramides also trigger caspase-independent death pathways. In ovarian cancer models, ceramides induce necroptosis through direct MLKL activation, promoting its oligomerization and relocalization to the plasma membrane. This process occurs independently of RIPK1 and RIPK3, culminating in membrane rupture and regulated necrotic death (Zhang et al., 2018).

Autophagy is a cellular process for degrading and recycling components to maintain homeostasis. This study shows ceramide promotes autophagosome formation by enhancing LC3 lipidation, tipping cells toward excessive autophagy and autophagy-dependent cell death (Autosis) under amino acid deprivation (Nah et al., 2020; Taniguchi et al., 2025) Haga clic o pulse aquí para escribir texto. In head and neck squamous cell carcinoma and murine embryonic fibroblasts, C18-ceramide generated by CerS1 induces lethal mitophagy through direct interaction with LC3B-II on mitochondrial membranes, dependent on mitochondrial fission and culminating in caspase-independent cell death (Sentelle et al., 2012).

Beyond acute cell death, ceramides participate in sustained cellular stress responses. Endoplasmic reticulum stress, arising from altered Ca2+ homeostasis and accumulation of misfolded proteins, activates caspase-mediated apoptotic pathways, linking sustained stress to regulated cell death. In human post-mortem brain tissue from Alzheimer’s disease patients, insulin/IGF resistance associates with increased ceramides and sustained ER stress activation, favoring inflammation, cytoskeletal disruption, Aβ secretion, and neurodegenerative progression that amplifies with disease severity (de la Monte et al., 2012).

Together, ceramides emerge as central regulators of regulated cell death and endoplasmic reticulum stress. However, direct evidence in MDD remains scarce, underscoring the need for studies examining ceramide effects on microglia, synaptic function, and ER stress using preclinical and translational depression models.

### 4.2 Ceramides and mitochondrial signaling

Ceramide accumulation drives lipotoxicity in non-adipose tissues, disrupting mitochondrial homeostasis by impairing ATP production, oxidative stress regulation, mitochondrial number, and quality control. This review highlights ceramides’ central role in apoptosis, differentiation, and mitochondrial dysfunction, guiding future research directions (Ding et al., 2024).

As mentioned above, Ceramide critically regulates apoptosis. This by targeting mitochondria, promoting mitochondrial outer membrane permeabilization (MOMP) (Fisher-Wellman et al., 2021). Also, we mentioned, Ceramides regulates autophagy. Ceramides play a central role in mitochondria-to-nucleus signaling by integrating with autophagy, mitophagy, and TOR pathways. Ceramide synthase genes, such as yeast LAG1 and worm orthologs hyl-1/hyl-2, regulate lifespan, linking ceramide signaling to cellular quality control and cytoprotective mechanisms (Jazwinski, 2015). Ceramides disrupt mitochondrial function in neurons by altering membrane composition, inhibiting the respiratory chain, increasing ROS and oxidative stress, and activating mitophagy. These bioactive lipids contribute to energy imbalance and neurodegeneration, while interventions like intermittent fasting can modulate ceramide metabolism to improve mitochondrial health (Valenzuela-Ahumada et al., 2025).

Indirectly, direct evidence shows that patients with MDD present reduced glucose metabolism. Adult patients with MDD (188 cases against 169 healthy controls). Through voxel based meta analysis using ALE software on 10 whole brain FDG PET studies, cerebral glucose metabolism was evaluated. Bilateral hypometabolism was detected in the insula, putamen, caudate nucleus and cingulate gyrus, and hypermetabolism in the thalamus, cerebellum and vermis. Altered mitochondrial metabolism in these limbic cortical subcortical regions suggests neuronal energetic dysfunction as a central pathophysiological substrate of MDD (Su et al., 2014). In other previous studies it had already been demonstrated in basal ganglia that 35 adults with unmedicated MDD against 18 controls showed mitochondrial β ATP reduced 16% in depressed subjects, evidencing a direct deficit of mitochondrial high energy phosphorylative metabolism in MDD (Moore et al., 1997). More recently, 40 adult women (mean age 46.1 years) with unmedicated melancholic depression were compared against 20 healthy controls (49 years) to evaluate the inflammatory effect and the role of mitochondrial biogenesis using gene expression in blood monocytes (qPCR). Downregulation of PGC-1α, TFAM and NRF1 was detected (p < 0.001), reduction of ATP, increase of mitochondrial ROS and inflammatory overactivation (IL-6, TNF-α, NLRP3). The deficit of mitochondrial biogenesis compromises oxidative phosphorylation, establishing it as a central pathophysiological mechanism and potential therapeutic target in MDD. BDI elevated (41.2 ± 5.9 against 4.9 ± 3.2, p < 0.001). Antioxidants CuZnSOD and MnSOD decreased (p < 0.001), confirming systemic mitochondrial energetic dysfunction. The deficit of mitochondrial biogenesis compromises oxidative phosphorylation, establishing it as a central pathophysiological mechanism and potential therapeutic target in MDD (Alcocer‐Gómez et al., 2016).

Regarding anaerobic glycolysis, 19 adults with MDD (31.7 years, unmedicated) against 20 controls (28.1 years). Using 1H MRS spectroscopy (3T, J resolved PRESS) in the anterior cingulate prefrontal cortex, elevated glucose (2.0 against 1.2 mmol/L, p = 0.043) and increased lactate (1.6 against 1.0 mmol/L, p = 0.009) were detected, with positive glucose lactate correlation (r = 0.72, p = 0.027). The pattern of increase in both reflects mitochondrial inability to oxidize glucose through OXPHOS, evidencing compensatory anaerobic glycolysis as a signature of mitochondrial dysfunction in MDD (Ernst et al., 2017).

In the respiratory chain, previous studies evaluated effects in bipolar disorder, schizophrenia and MDD, highlighting specifically MDD. Fifteen adults with *post mortem* MDD were evaluated against 15 controls (matched by age, sex, brain pH). Through RT PCR and immunoblotting in the lateral cerebellum, a specific reduction of subunits of NADH ubiquinone oxidoreductase (Complex I), the initial enzyme of the mitochondrial respiratory chain, was detected. It was found that NDUFV1 decreased 57%, NDUFV2 decreased 47%, NDUFS1 decreased 36% (p < 0.05), with proteins concordantly reduced (49%, 45%, 32%, p ≤ 0.001). In the striatum, MDD did not differ from controls, evidencing mitochondrial OXPHOS dysfunction specifically in cerebellum in MDD. Interestingly, schizophrenia showed the opposite dysfunction with NDUFV1 reduced in striatum (not cerebellum), while bipolar disorder presented partial involvement in both regions (Ben-Shachar and Karry, 2008) Mitochondrial neuronal protein levels have also been reported in MDD. Twenty adults with unmedicated MDD (age 32 years) against 10 healthy controls (28 years). Through ELISA in plasma neuronal extracellular vesicles (NDEVs L1CAM+), 14 mitochondrial proteins were quantified. In basal MDD it was observed that Complex I decreases (374 against 1852 pg/mL, p < 0.0001), while Complex III decreases, NMNAT2 decreases, SARM1 increases, MFN2 decreases, MOTS c decreases (p < 0.0001). Responders to SSRI normalized Complex III, MFN2 and MOTS c, suggesting that reversible neuronal energetic mitochondrial dysfunction exists as a biomarker in MDD (Goetzl et al., 2021).

In animal models of adult C57BL/6J mice (10–12 weeks) with chronic social defeat stress (CSDS), it was demonstrated that the deficit of astrocytic release in medial prefrontal cortex and hippocampus of ATP as a direct marker of energetic mitochondrial dysfunction induced depressive behaviors. Administration of ATP (125 mg/kg) reversed these behaviours in 7 days, surpassing the efficacy of imipramine (p < 0.001), indicating mitochondrial purinergic energetic metabolism as a therapeutic target in MDD (Cao et al., 2013).

Regarding evidence that directly relates mitochondria and suicide. It was found that mitochondrial senescence in depressed adolescents with suicidality, in one study including 53 adolescents (16–19 years). Of these, 19 had MDD with suicide attempt or ideation (MDD + SA/SI), 14 had MDD without suicidality and 20 were healthy controls. In genomic DNA from peripheral blood, telomere length (TL) and mitochondrial DNA copy number (mtDNAcn) were measured by qPCR. Total MDD showed decreased TL (p = 0.045) and increased mtDNAcn (p = 0.028) compared with controls. The MDD + SA/SI subgroup showed more pronounced changes (p = 0.024 and p = 0.022). mtDNAcn positively correlated with childhood emotional abuse (r = 0.29, p = 0.025), suggesting that early stress accelerates mitochondrial dysfunction as a mechanism of cellular senescence in adolescent MDD (Ochi et al., 2023).

On the other hand, another study evaluated the role of possible mitochondrial genetic biomarkers of suicide in MDD in blood and prefrontal cortex in 24 adults with MDD suicide (∼42 years) and 24 with MDD non suicide (∼51 years) against 21 controls. In post-mortem blood and DLPFC, using NanoString nCounter (114 genes), MTPAP (mitochondrial poly A polymerase) increases (p = 0.002, FC = 1.801) and SLC25A26 (mitochondrial metabolite transporter) increases (p = 0.006, FC = 2.119) in MDD suicide compared with MDD non suicide, evidencing alteration of mitochondrial RNA metabolism and transport of energetic substrates as specific biomarkers of suicide in MDD (Mamdani et al., 2022) Indirectly this relates mitochondria, suicide and ceramide, since SLC25A26 transports S adenosylmethionine (SAM) toward the mitochondria, which is a methyl group donor for sphingomyelin synthesis and ceramide metabolism through methylation. A deficiency of mitochondrial SAM could alter the ceramide sphingomyelin balance, which has been associated with neuronal apoptosis and depression (Marí et al., 2004; Guo et al., 2024) Additionally, another indirect evidence is a study evaluating metabolic mitochondrial dysfunction in refractory MDD with suicidal ideation in 99 adults with trMDD SI (median 29 years, onset ∼13 years) against 93 healthy controls. In plasma (LC MS MS, 672 metabolites, ELISA for FGF21 and GDF15), mitochondrial dysfunction was identified since FGF21 increases in both sexes, GDF15 increases in women (p < 0.05), lactate increases and cystine decreases, indicating reductive stress due to reduced mitochondrial oxidation of NADH. Ceramides and sphingomyelins decrease in both sexes. CoQ10 decreases individually, confirming multisystemic mitochondrial energetic deficit in treatment resistance MDD SI (Pan et al., 2023). The increase of FGF21 is important since FGF21 reduces ceramide synthesis. First it stimulates adiponectin and this activates ceramidases that decrease accumulation of intracellular ceramides (Holland et al., 2013).

### 4.3 Ceramide chain-length balance regulates cell function inflammation and mood

Ceramides do not constitute a homogeneous entity but rather a family of sphingolipids whose structural diversity determines differentiated biological functions. This variability, particularly in acyl chain length and saturation, is conferred primarily by ceramide synthases (CerS1-6), each with preference for fatty acids of different lengths, generating ceramide species with tissue-specific and subcellular profiles (Mullen et al., 2011).

Short-chain ceramides reduce lipid packing, disrupt the long periodicity phase (LPP), and increase membrane permeability, whereas, Long-chain ceramides maintain LPP integrity, dense lipid packing, and proper barrier function (Uche et al., 2021).

Long and very long chain ceramides, primarily generated by ceramide synthase 2 (CerS2), support normal cell physiology by maintaining a balance with shorter ceramides, promoting cell proliferation, growth, and homeostasis. In contrast, short-chain ceramides induce apoptosis and inhibit proliferation. Thus, equilibrium between long and very long ceramides is crucial for healthy cellular function (Hartmann et al., 2012).

Patients with Inflammatory Bowel Disease (IBD) and Primary Sclerosing Cholangitis (PSC) have elevated long-chain (LC) ceramides and reduced very long-chain (VLC) ceramides, resulting in a higher LC/VLC ratio correlated with systemic inflammation (CRP, calprotectin, cholestasis markers). This LC/VLC imbalance may indirectly contribute to MDD by promoting neuroinflammation, disrupting hippocampal lipid signalling, and impairing mood-regulating pathways, linking peripheral inflammation with depressive (Elger et al., 2025).

### 4.4 Ceramides, peripheral lipid dysfunction, and systemic inflammation

Beyond their role in intracellular communication, circulating ceramides constitute a functional axis linking peripheral metabolism to systemic inflammation and central nervous system function.

Specific ceramide species can cross the blood-brain barrier and exert direct central effects. Short-chain ceramides such as C2 are associated with insulin resistance states and, upon crossing the BBB, inhibit insulin/IGF-1 receptors, IRS-1, and Akt, contributing to neurodegeneration and cognitive deficit (de la Monte et al., 2010). Circulating ceramides also activate peripheral immune cells: in human monocytes, ceramide-enriched LDL activates TLR4/CD14 and induces IL-6, IL-10, and MCP-1 release, demonstrating that ceramides can convert LDL into an innate proinflammatory stimulus (Estruch et al., 2014).

Clinical interpretation of ceramide alterations in MDD is complicated by metabolic confounders including BMI, diet, diabetes, and drug use, which independently affect both ceramide levels and endothelial function. In human tibial arteries from diabetic individuals, C16 ceramide was elevated 11% and correlated with sphingomyelin, while increased SMPD4 expression (without changes in CERS6) associated with vascular damage. In HUVECs, C16 reduced proliferation, increased apoptosis, and decreased autophagy, indicating a causal role of C16/SMPD4 in diabetic endothelial dysfunction (Meade et al., 2023). Such endothelial alterations may impact blood-brain barrier integrity, potentially facilitating ceramide access to the CNS.

Despite these confounders, evidence supports ceramide involvement in depression specifically. In a cross-sectional study of 25 controls and 21 Alzheimer’s patients, recent major depression was associated with significant increases in plasma ceramides (C16:0, C18:0, C20:0, C24:1, C26:1) with large effect sizes (d = 0.73–2.39), independent of Alzheimer’s diagnosis, antidepressant use, or GDS scores, suggesting ceramides as peripheral biomarkers of active depression (Gracia-Garcia et al., 2011). Mechanistic support comes from a murine chronic stress model, where stress increased plasma exosomes enriched in nSMase2-dependent ceramides. Transfer of these exosomes induced depressive behaviors (anhedonia, immobility) by inhibiting hippocampal endothelial PLD and reducing phosphatidic acid; nSMase2 deficiency or ceramide neutralization prevented these effects (Schumacher et al., 2022).

### 4.5 Ceramides modulate astrocytes and blood-brain barrier integrity in neuroinflammation and depression

The transmission of inflammatory and metabolic signals to the brain depends on the blood-brain barrier (BBB). This dynamic, selective structure, formed by endothelium, basement membrane, and junctions, is regulated by astrocytes via released factors, extracellular vesicles, transcriptional and non-coding mechanisms, and structural control of tight and adherens junctions, basement membrane, and endothelial cytoskeleton (Manu et al., 2023).

Ceramides directly modulate astrocyte function and BBB properties. In rat cerebellar astrocytes, long-chain ceramides generated by sphingosine recycling and acid sphingomyelinase predominate; bFGF reduces ceramide levels by activating sphingomyelin synthase, favoring proliferation, while ceramide inhibits astrocyte growth by blocking the MAPK pathway (Riboni et al., 2002). In Parkinson’s disease dementia (PDD), and frontotemporal lobar dementia (FTLD) post-mortem tissue, neuroinflammation correlated with increased proapoptotic short- and medium-chain ceramides in astrocytes, linked to elevated CerS5 expression. HPLC-MS/MS confirmed these lipid shifts, indicating a shared ceramide-mediated mechanism driving neuroinflammatory damage and astrocyte dysfunction across dementias (Manu et al., 2023) Altered ceramide profiles may disrupt BBB integrity and promote neuroinflammation, indirectly contributing to MDD pathophysiology.

Sustained systemic inflammation compromises BBB integrity by affecting cerebral endothelial cells, disorganizing tight junctions, and increasing permeability to potentially neurotoxic peripheral molecules and cells (Brandl and Reindl, 2023). Proinflammatory cytokines and circulating bioactive lipids induce endothelial oxidative stress and inflammatory pathway activation, rendering the BBB functionally vulnerable. In Wistar rats on high-fat diet, alpha-lipoic acid reduced ceramide synthesis, oxidative stress, and brain inflammation while decreasing apoptosis and β-amyloid accumulation and improving systemic insulin resistance through redox and metabolic modulation (Klandorf and Dartigue, 2024; Maciej et al., 2022). The increase in circulating ceramides, as seen in fatty liver disease, may indirectly promote the production of neurotoxic ceramides in the liver and possibly in adipocytes in obesity, type 2 diabetes, or NASH. These circulating ceramides can cross the blood–brain barrier, partially explaining the co-occurrence of cognitive impairment and neurodegeneration in these conditions (Tong and de la Monte, 2009).

Circulating ceramides and sphingolipids can directly impact cerebral endothelium, modulate BBB integrity and facilitate passage of immune and metabolic mediators. In isolated rat brain capillaries, ceramide-1-phosphate rapidly and reversibly increased P-glycoprotein activity without altering expression, an effect dependent on the COX-2/prostaglandin E2 pathway, demonstrating dynamic sphingolipid regulation of BBB permeability (Mesev et al., 2017). Notably, certain ceramides can cross the BBB directly, where, in a murine Alzheimer’s model using *in vivo* and *in vitro* BBB systems, plant-type d18:2 ceramides crossed the barrier, were partially metabolized to sphingomyelin and GlcCer, and reduced brain amyloid, demonstrating functional peripheral-to-central access (Eguchi et al., 2020).

Direct evidence in MDD remains limited. However, findings from inflammatory and neurodegenerative conditions document increased immunometabolic trafficking to the brain, suggesting that the BBB acts as an active modulator of peripheral signal propagation rather than a static barrier, potentially contributing to chronic exposure of brain parenchyma to inflammatory mediators in depression.

### 4.6 Ceramides influence microglial priming and neuroinflammation in brain function and depression

Ceramides and sphingolipids are particularly abundant in the brain, inhibition of ceramide biogenesis with myriocin or fumonisin B1 reduces neural progenitor motility (Chinnappa et al., 2025). Persistent brain exposure to peripheral inflammatory signals induces functional changes in microglia, particularly a priming state characterized by exaggerated responses to secondary stimuli and greater proinflammatory cytokine release without requiring intense initial activation (Lima et al., 2022).

Bioactive lipids participate in this process with context-dependent effects. In primary microglia stimulated with LPS *in vitro* and in mice with carotid exposure under general anesthesia, C8-ceramide increased BDNF and improved cognition via PKCδ/NF-κB signaling, demonstrating a neuroprotective effect (Li G. et al., 2024). However, lipid-mediated priming can also be detrimental: intracerebroventricular neuraminidase administration induced acute neuroinflammation and microglial priming in mice, with hypothalamic microglia showing exacerbated responses to high-fat diet or LPS at 3 months, accompanied by microgliosis, POMC neuron reduction, increased intake, and paradoxical body weight decrease (Fernández‐Arjona et al., 2022).

Importantly, BBB disruption is not required for peripheral inflammation to impact microglial function and behavior. In murine social defeat models, peripheral inflammation induced monocyte trafficking, microglial activation, and priming without BBB rupture; CSF1R antagonist inhibition blocked myeloid recruitment and attenuated anxiety-like behavior (Dantzer, 2019).

Specific evidence in MDD remains limited, though the role of neuroinflammation has been extensively reviewed. These findings highlight the need for basic research examining whether microglial priming constitutes a functional bridge between systemic inflammation and brain dysfunction in depression.

### 4.7 Synaptic alterations, plasticity, and mood circuits

Synaptic plasticity constitutes a central mechanism through which the nervous system adapts to environmental stimuli and experiences. This process depends on coordinated interaction between glutamatergic AMPA and NMDA receptors. NMDA activation allows postsynaptic Ca2+ entry, triggering cascades that regulate AMPA receptor insertion or removal and processes of long-term potentiation and depression (LTP and LTD) (de León-López et al., 2025). In post-mortem brains of 10 MDD, 10 bipolar disorder, and 10 control subjects, [^3^H] AMPA and [^3^H] kainate binding measured glutamate receptor levels. MDD subjects showed a 20.7%–27.7% increase in [^3^H] AMPA binding in the anterior cingulate (BA 24) but not dorsolateral prefrontal cortex (BA 46). No changes were observed in bipolar disorder or for [^3^H] kainate binding in either region for both disorders, indicating region- and receptor-specific alterations in MDD (Gibbons et al., 2012) In MDD specifically, single-nucleus RNA-seq analysis identified excitatory neuron subpopulations (Excitatory.neurons_1 cells) with altered CASKIN1/CSTB hubs and miR-21-5p-mediated CASKIN1 repression, linking disrupted synaptic plasticity with reduced connectivity, possibly in a male-specific manner (Chen et al., 2025).

In addiction models, aberrant plasticity in the nucleus accumbens consolidates persistent associations with rewarding stimuli, while experimental LTD induction reverses reward anxiety and reduces relapse, positioning synaptic plasticity as a therapeutic target in psychiatry (Ramirez and Arbuckle, 2016).

Inflammation and bioactive lipids directly modulate synaptic plasticity through ceramide-dependent mechanisms. In cultured neurons, TNFα activates nSMase2, increasing ceramides and promoting NMDA receptor synaptic trafficking, NR1 phosphorylation and clustering, elevated Ca2+, and potentiation of excitatory postsynaptic currents; nSMase2 inhibition abolished these effects, demonstrating an inflammation-dependent lipid mechanism (Wheeler et al., 2009).

These findings have therapeutic implications. Emerging glutamatergic therapies for treatment-resistant depression, affecting approximately 30% of MDD patients, converge on NMDA or AMPA signaling to potentiate AMPA receptors, BDNF-mTOR pathways, synaptic strengthening, receptor trafficking, and spine formation, positioning these agents as next-generation antidepressant strategies (Freudenberg et al., 2025).

### 4.8 Human evidence of altered ceramides and lipid profiles in MDD and TRD

Lipidomic and metabolomic characterization in peripheral blood has enabled identification of specific ceramide profiles associated with major depression (MDD) and other affective disorders. In a study on adolescents (n = 112), UPLC-MS/MS was used after lipid extraction to analyze plasma, identifying specific ceramides, such as Cer (18:0/15:0), Cer (18:1/16:0), Cer (18:1/26:0), Cer (18:0/22:0), Cer (d18:0/18:1), and CerP (18:1/18:0), that distinguished with high discrimination between control, PPP, and CPP, with AUC of 0.938–0.964 (Nguyen et al., 2024).

In another study, 67 patients with unipolar or bipolar disorder and 405 healthy controls mached by gender, age and BMI, targeted and untargeted lipidomic analysis identified elevations of C16-C24:1 ceramide and hexosylated metabolites (C24:1GluCer, C24LacCer), especially in males, correlating with age, triglycerides, and antidepressant use, independently of episode or MADRS improvement (Brunkhorst-Kanaan et al., 2019).

A study in Han ethnicity women with MDD and BPD in depressive episode, using UHPLC-MS/MS, revealed alterations in sphingolipids, glycerophospholipids, and acylated fatty acids, identifying 20 key lipid species as combinational biomarkers of MDD, 8 for BPD, and 13 to differentiate both disorders, correlating with clinical severity assessed by HAMD, HAMA, and PANSS (Zhang et al., 2022). Likewise, an analysis of 107 MDD patients and 97 healthy controls with UHPLC-Q-Exactive HF MS showed significant differences in 40 lipids, including oxidized fatty acids and altered acylcarnitines, with OxFAs being the most discriminative for MDD (He et al., 2025).

Collectively, these findings support the utility of peripheral lipidomic profiling for identifying candidate MDD biomarkers, although whether these associations reflect causal mechanisms or epiphenomena requires further investigation.

### 4.9 Patterns associated with severity and clinical subtypes

The clinical heterogeneity in MDD and TRD, manifested in affective, cognitive, and somatic symptoms, is paralleled by heterogeneity in lipid profiles (see Table 1). Lipidomic studies have identified specific ceramides (C16-C24:1Cer) and hexosylated metabolites that distinguish clinical subtypes and correlate with severity, particularly in men (Brunkhorst-Kanaan et al., 2019), while in women with MDD and BPD, combinations of sphingolipids, glycerophospholipids, and fatty acids relate to symptom intensity and allow differentiation of disorders (Zhang et al., 2022).

**TABLE 1:** Ceramide alterations in major depressive disorder and their association with clinical severity.

Major depressive disorder (MDD)						
Author/Year [Ref]	Study Design	Population, Matrix and Platform	Ceramide Findings and Severity Correlation	Treatment Response and Suicidal Ideation	Confounders Controlled and Main Limitations	Biomarker Potential
Gracia-Garcia et al. (2011)	Cross-sectional; sub-analysis by recent/past depression	Older adults ≥55 years; NC n = 25, AD n = 21 Recent/past/no depression subgroups Plasma (non-fasting) · LC-MS/MS (MRM)	↑ Cer C16:0, C18:0, C20:0, C24:1, C26:1 in recent depression (≤2 years) vs. no/past depression C22:0, C24:0: NS. No correlation with GDS.	Not assessed; no ceramide differences by antidepressant use (n too small)	No group differences in age/sex/education; no multivariable model Limitations: Self-report diagnosis (no structured interview); small n; broad ‘recent’ window (≤2 years)	Medium — biologically consistent signal; high risk of bias (diagnosis quality, sample size)
Brunkhorst-Kanaan et al. (2019)	Observational case-control; targeted lipidomics; multivariable analyses	Unipolar/bipolar n = 67 vs. HC n = 405; sex/age/BMI matched Plasma · Targeted + untargeted lipidomics; tandem MS	↑ C16–C24:1 Cer; ↑ C24:1GluCer, C24LacCer (stronger in men) Not associated with current episode status	Not associated with MADRS improvement; antidepressant use → higher ceramides (not response)	Matching sex/age/BMI; triglycerides/diacylglycerols and medication as covariates Limitations: Mixed MDD + BD; pharmacological confounding; no predictive treatment value	Medium — large HC group; limited psychiatric specificity; no treatment-response prediction
Schumacher et al. (2022)	Case-control + murine chronic stress model (mechanistic arm)	HC n = 16 vs. untreated MDD n = 16 (mod.–severe) Plasma (human); plasma exosomes + hippocampal tissue (mice) Ceramide sum C16–C24; nSMase2 assays	↑ Plasma ceramides in MDD vs. HC. Ceramide-enriched exosomes (nSMase2-dependent) transfer depressive behavior in mice; inhibition reverses effects Correlated with HAM-D	nSMase2 inhibition reverses depressive behavior in mice (preclinical therapeutic implication). Not assessed in humans (untreated cohort)	ANCOVA: BMI/age/sex; groups comparable on clinical characteristics Limitations: Small human n; aggregated ceramide measure (C16–C24 sum); no longitudinal validation	Medium-high — state/severity signal with confounder control; nSMase2-ceramide-exosome axis as therapeutic target; replication needed
Zhang et al. (2022)	Cross-sectional case-control; targeted lipidomics	Adult Han women; MDD (depressive episode), BD, and HC Plasma · UHPLC-MS/MS (targeted + untargeted)	20 lipid species for MDD; 8 for BD; 13 for MDD vs. BD differentiation Sphingolipids, glycerophospholipids, acylated FAs Correlated with HAMD, HAMA, PANSS.	Not assessed	Sex and ethnicity homogeneous (Han women); clinical scales applied systematically Limitations: Single sex/ethnicity; cross-sectional; medication status not fully reported	Medium — multi-lipid biomarker candidates with severity correlation; requires replication in mixed-sex/multi-ethnic cohorts
He et al. (2025)	Case-control; untargeted lipidomics	MDD n = 107 vs. HC n = 97 Plasma · UHPLC-Q-Exactive HF MS	40 dysregulated lipid species; OxFAs and acylcarnitines most altered (strongest discriminative capacity). Ceramide-specific signal not isolated	Not assessed	Matched case-control; high-resolution platform Limitations: Cross-sectional; ceramide species not highlighted; clinical confounders not fully detailed	Medium — OxFAs/acylcarnitines as complementary lipid biomarkers; ceramide-specific signal requires isolation
Dinoff et al. (2017)	Systematic review	Human studies; depressive adults; n varies by study Peripheral blood (plasma/serum) Multiple targeted ceramide panels	C18:0 and C20:0 consistently ↑ in depression C16:0: Inconsistent across studies Formal correlation with severity: Variable	Not assessed	Confounder control variable across included studies Limitations: Heterogeneity in diagnostic criteria, platforms, and covariate adjustment; limited studies	Medium — first systematic evidence; establishes C18:0 and C20:0 as priority ceramide species
Demirkan et al. (2013)	Observational; community-based family lipidomics study	Dutch family-based population sample (adults); n not specified in manuscript Plasma · lipidomics (phosphatidylcholine and sphingomyelin panels; MS-based)	Plasma phosphatidylcholine and sphingomyelin concentrations significantly associated with depression and anxiety symptoms; ceramide-related sphingolipid alterations identified in community-based sample	Not assessed	Community-based design increases external validity vs. clinical cohorts; family-based structure controls for shared genetic variance Limitations: ceramide species not individually specified; cross-sectional; anxiety and depression symptoms not fully separated	Medium — extends ceramide/sphingolipid-depression association to community populations; supports generalizability beyond clinical cohorts
Vega-Beyhart et al. (2021)	Observational metabolomics; Cushing syndrome model of sustained hypercortisolemia	Cushing syndrome patients + HC; n NR Plasma · untargeted metabolomics; MS-based lipid profiling	93 altered metabolites incl. ceramides (species NR), TAGs, GPLs, cholesterol esters Urinary cortisol predicted multiple lipid concentrations (indirect HPA-ceramide link)	Not assessed	Urinary cortisol measured independently; Kennedy pathway and Lands cycle disruptions documented Limitations: Extreme cortisol model; direct extrapolation to MDD/TRD requires caution; ceramide species not individually specified	Indirect/mechanistic — supports HPA-ceramide metabolic axis; no direct clinical ceramide biomarker data
Elger et al. (2025)	Observational cross-sectional; IBD and PSC cohort	Adults with IBD and PSC; controls; n NR Peripheral blood · targeted ceramide panel; MS	↑ LC ceramides; ↓ VLC ceramides ↑ LC/VLC ratio correlated with CRP, calprotectin, and cholestasis markers	Not assessed	CRP and calprotectin as severity References; cohort defined by established clinical diagnoses Limitations: Non-psychiatric cohort; causal direction not established; indirect extrapolation to MDD/TRD	Indirect/mechanistic — LC/VLC ceramide imbalance as systemic inflammatory marker; supports peripheral ceramide dysregulation hypothesis in MDD

These findings must be interpreted cautiously given the discrepancy between DSM-5 and ICD-10 criteria and the inherent variability in biomarker measurement, underscoring the need to standardize diagnostic definitions and analytical methods. Integrating lipid profiles with clinical criteria and functional neuroimaging data, such as interhemispheric connectivity associated with suicidal ideation (Wang et al., 2025), may enable identification of reproducible severity patterns and biological subtypes. Such multimodal approaches combining clinical, metabolic, and neurobiological measures offer a path toward overcoming diagnostic heterogeneity and improving risk stratification for personalized interventions.

### 4.10 Ceramides and treatment outcomes

Evidence linking ceramides to treatment response in MDD remains incipient. Preclinical studies demonstrate that plasma exosomes enriched in nSMase2-generated ceramides induce depressive behaviors in murine chronic stress models by inhibiting hippocampal phospholipase D and reducing phosphatidic acid; neutralization of ceramides or nSMase2 deficiency prevented these effects, suggesting a direct role in behavior modulation with potential therapeutic implications (Schumacher et al., 2022).

In humans, clinical studies have not yet directly evaluated ceramide-based prediction of treatment response. However, certain long-chain ceramide species correlate with antidepressant use rather than clinical improvement, suggesting their elevation may reflect pharmacological exposure rather than therapeutic efficacy (Brunkhorst-Kanaan et al., 2019). This raises the possibility that specific lipid profiles could be integrated into future stratification approaches, particularly for therapies based on glutamatergic modulation targeting AMPA-NMDA signaling, BDNF, and mTOR pathways (Freudenberg et al., 2025).

Collectively, these findings suggest a link between ceramides and molecular mechanisms underlying synaptic plasticity and inflammatory signaling that could influence treatment efficacy. However, human evidence remains limited, and longitudinal translational studies are needed to validate ceramides as biomarkers of therapeutic response.

### 4.11 Acid sphingomyelinase as a central regulator of ceramide signaling and a therapeutic target in MDD

Acid sphingomyelinase (ASMase) hydrolyzes the cleavage of the phosphocholine head group of sphingomyelins to generate ceramide (Ze et al., 2010), a key signaling lipid regulating cell death, ER stress, autophagy.

Aberrant ASMase–ceramide signaling contributes to multiple hepatic pathologies, including steatohepatitis, fibrosis, and hepatocellular carcinoma, highlighting ASMase as a promising therapeutic and drug-repurposing target (Mir and Thirunavukkarasu, 2023). Acid sphingomyelinase (ASMase) generates ceramide, modulating membrane structure and signaling beyond lipid metabolism. Distinct ASMase forms and cellular localizations enable macrophage functions and contribute to multiple diseases driven by macrophage activation (Truman et al., 2011).

This enzyme has been shown to exert a dual role in sepsis, where, SMPD1 activation drives ceramide generation, lipid raft formation, receptor clustering, cytotoxicity, and ADAMTS13 downregulation, while its pharmacological or genetic inhibition mitigates endothelial damage and improves stress responses, highlighting ASMase as a potential therapeutic target (Chung et al., 2016).

L-SMase–driven ceramide synthesis supports cellular homeostasis, whereas S-SMase–derived ceramides preferentially amplify inflammatory signaling pathways. The SMPD1 gene encodes two distinct acid sphingomyelinase isoforms with divergent functions. Lysosomal sphingomyelinase (L-SMase) degrades sphingomyelin within lysosomes and is essential for maintaining tissue lipid homeostasis. Secretory sphingomyelinase (S-SMase), in contrast, is released into circulation and contributes to inflammatory and chemokine signaling. Experimental models show that preserving L-SMase while eliminating S-SMase activity prevents sphingomyelin accumulation, neurodegeneration, and motor impairment in Niemann–Pick disease, underscoring compartment-specific biological roles (Beard et al., 2025).

Recent studies link MDD to increased acid sphingomyelinase (ASM) activity. Many antidepressants act as functional ASM inhibitors (FIASMAs) but induce lysosomal phospholipids. This study shows that minor chemical modifications of FIASMAs can enhance ASM inhibition while reducing phospholipidosis, highlighting a strategy to develop safer, more targeted antidepressants (Rhein et al., 2018).

The most common antidepressants FIASMA including amitriptyline, imipramine, desipramine, fluoxetine, sertraline, escitalopram, and maprotiline, inhibited ASM and the formation of ceramide-enriched membrane (Hoertel et al., 2022).

### 4.12 Common methodological limitations

Lipidomic studies in MDD and TRD show consistent alterations in ceramides and related metabolites; however, these findings are constrained by substantial methodological limitations. Sample heterogeneity, including variability in age, sex, number of depressive episodes, antidepressant exposure, and comorbid conditions, complicates cross-study comparisons (Brunkhorst-Kanaan et al., 2019) In addition, the lack of standardization in lipid extraction procedures, analytical platforms, and data normalization methods, together with the use of diverse clinical scales to assess symptom severity and depressive subtypes, limits reproducibility and the generalizability of reported lipid profiles (Nguyen et al., 2024; He et al., 2025). Moreover, the predominance of cross-sectional study designs precludes the establishment of causal relationships between ceramide alterations and clinical outcomes. Collectively, these limitations underscore the need for harmonized methodological protocols, the inclusion of more representative samples, longitudinal study designs, and integration with functional and neurobiological biomarkers to generate robust and clinically translatable findings.

### 4.13 Ceramides as emerging biomarkers of suicidal behavior

Suicidal behavior is a major cause of injury and mortality worldwide. It shows a higher incidence of death in men and a greater frequency of non-fatal attempts in women, young individuals, single people, and patients with psychiatric disorders, despite the overall increase in therapeutic interventions (Nock et al., 2008).

Studies in patients with MDD report markedly elevated odds ratios for suicidal ideation, including lifetime (OR = 2.88), last-month (OR = 49.88), and last-year estimates (OR = 13.97). Increased risks have also been observed for suicidal planning (OR = 9.51) and suicide attempts, both over the lifetime (OR = 3.45) and in the previous year (OR = 7.34). In contrast, no significant differences have been reported for completed suicide (OR = 0.69), highlighting the importance of early screening and continuous clinical monitoring (Cai et al., 2021).

In younger populations aged 11–24 years, MDD is associated with a significantly higher risk of suicidal ideation (OR = 3.89). This association appears stronger in Asian populations compared with those in the Americas (OR = 4.71 compared to 1.71) and is more pronounced in younger adolescents and in earlier studies (Souza et al., 2023). Overall, these findings support the presence of an elevated suicide risk in individuals with MDD and emphasize the relevance of early preventive interventions. However, direct evidence supporting ceramides as biomarkers of suicidal behavior in human populations remains limited (see Table 2).

**TABLE 2:** Biological correlates of suicide and suicidal ideation in major depressive disorder.

Suicide and suicidal ideation						
Author/Year [Ref]	Study Design	Population, Matrix and Platform	Ceramide Findings and Severity Correlation	Treatment Response and Suicidal Ideation	Confounders Controlled and Main Limitations	Biomarker Potential
Cai et al. (2021)	Systematic review and meta-analysis (15 studies)	MDD vs. non-depressed; pooled n = 85,768 Epidemiological · not applicable	Ceramides not reported	Significantly ↑ odds for suicidal ideation (monthly OR = 49.88; annual OR = 13.97; 2-week OR = 24.81), planning (OR = 9.51), attempts (OR = 3.45) Completed suicide: OR = 0.69 (NS)	Pooled analysis; statistical heterogeneity assessed Limitations: Methodological heterogeneity; completed suicide OR NS (possible ascertainment bias)	Epidemiological — establishes MDD as high-risk context for suicidality; does not address ceramide mechanisms
Dold et al. (2018)	Observational cross-sectional; GSRD European multicenter study	MDD patients n = 1,410 from GSRD; suicidality measured by HAM-D item 3 Clinical database · standardized psychiatric assessment; no ceramide platform	Ceramides not reported; 46.67% of patients exhibited some degree of suicidality; severe suicidality in 8.23%	Severe suicidality associated with inpatient treatment, antipsychotic/benzodiazepine use, melancholic features, and somatic comorbidities	Multicenter GSRD database; HAM-D item 3 as standardized suicidality measure Limitations: no ceramide or lipid biomarker measurement; cross-sectional; suicidality measure is a single item	Epidemiological — characterizes suicidality prevalence and severity correlates within MDD; provides clinical context for ceramide biomarker studies
Francis et al. (2025)	Post-mortem transcriptomics (RNA-seq + snRNA-seq deconvolution)	Post-mortem hippocampus: HC (C), MDD-no-suicide (D−S), MDD-suicide (D + S); n NR Brain (pyramidal neurons, microglia, endothelium) · RNA-seq + snRNA-seq	No ceramide measurement	Immuno-inflammatory activation (IL-10, IFN-γ; TLR2, CD86, CXCL8, ITGAM hubs) specific to D + S; absent in D−S	Three-group design isolates suicidal phenotype; cell-type-specific deconvolution Limitations: Post-mortem (agonal factors, PMI); no ceramide data; limited n	High mechanistic value — neuroinflammatory signatures specific to suicidal vulnerability within MDD; supports suicide-specific biomarker development
Mitro et al. (2020)	Prospective metabolomics study (antepartum cohort)	Pregnant women n = 100; antepartum depression and suicidal ideation outcomes Plasma · untargeted metabolomics; 307 metabolites (MS-based; broad Institute platform)	Nominal associations between suicidal ideation and ceramide species C24:1 and C24:0 No metabolite survived FDR correction Findings remain hypothesis-generating	Suicidal ideation assessed prospectively; ceramide species C24:1 and C24:0 showed nominal associations with suicidal ideation	Prospective design; broad metabolite coverage (307 metabolites); clinically defined suicidal ideation Limitations: Small n (n = 100); no FDR-corrected ceramide signal; pregnant women only — limited generalizability; ceramide findings unreplicated	High conceptual value — only human study reporting direct nominal ceramide-suicidal ideation associations (C24:1, C24:0); hypothesis-generating; requires replication in larger, adequately powered cohorts
Yang et al. (2025)	Observational cross-sectional	Adolescents with MDD + high suicide risk; n NR Peripheral blood · cytokine multiplex; KYN/TRP and 3-HK/KYN quantification	No ceramide measurement	KYN/TRP correlated with IL-1β, IL-6, IL-10, TNF-α; 3-HK/KYN with IL-8; TKP dysregulation linked to inflammation and suicide risk	Suicide risk stratification defined; cytokine and TKP metabolites co-measured Limitations: Cross-sectional; no ceramide data; n NR; adolescent findings may not generalize	Indirect — TKP-inflammation interface as biological correlate of suicide risk; relevant immunometabolic context
Ma et al. (2020)	Cross-sectional case-control	First-episode drug-naïve MDD n = 1,718; history of suicide attempts 20.14% Peripheral blood · Standard lipid panel (TC, LDL-c, HDL-c, TG); no ceramide platform	No ceramide measurement; ↑ TC, LDL-c, TG; ↓ HDL-c in attempters vs. non-attempters	↑ TC, ↓ HDL-c, and higher HAMD/HAMA independently associated with suicide attempts after adjustment	Adjusted for demographic and clinical variables; drug-naïve cohort removes medication confounding Limitations: no ceramide measurement; cross-sectional; retrospective attempt ascertainment	Indirect — pro-atherogenic lipid profile as biological risk marker; motivates ceramide-specific lipidomic studies in suicidal populations
Mamdani et al. (2022)	Case-control; blood transcriptomics and biomarker discovery	MDD patients with vs. without suicide (post-mortem and blood); adults Peripheral blood · RNA-seq; multi-omic biomarker panel; no direct ceramide platform	Ceramides not directly measured; multi-omic blood biomarker signatures identified distinguishing MDD with and without suicide	Blood-based gene expression and protein biomarkers associated with completed suicide in MDD; neuroinflammatory and metabolic pathways implicated	Multi-omic design; post-mortem and blood sample comparison Limitations: no ceramide measurement; completed suicide limits prospective validation; small n	High mechanistic value — multi-omic blood biomarker framework for suicide in MDD; supports integration of lipidomic/ceramide data into suicide biomarker panels
Wu et al. (2016)	Systematic review and meta-analysis (65 studies, 1980–2014)	Suicidal vs. non-suicidal patients + HC; pooled n = 510,392 Peripheral blood · Standard lipid panel; no ceramide platform	No ceramide measurement; ↓ TC, LDL-c, HDL-c, TG in suicidal patients	↓ TC associated with 112% ↑ in suicidality risk	Pooled analysis; subgroup analyses by suicidality type; heterogeneity assessed Limitations: no ceramide data; causal direction not established; heterogeneity across studies	Indirect — systemic lipid dysregulation as substrate for suicide risk; motivates ceramide-specific investigation
Park et al. (2024)	Retrospective cohort; sex-stratified	Suicide attempt patients n = 408 (adults) Peripheral blood (serum) · Standard lipid panel; no ceramide platform	No ceramide measurement; ↓ TC (<165 mg/dL) in women → ↑ suicide mortality	↓ TC in women independently associated with suicide mortality after attempt (HR 0.968–0.970)	Sex-stratified analysis; retrospective cohort with mortality endpoint Limitations: no ceramide data; small sex-stratified n; retrospective design	Indirect — sex-specific lipid vulnerability to suicide mortality; ↓ TC as potential risk indicator in women post-attempt
Daray et al. (2018)	Narrative review	Human studies; mixed adult populations; n varies Peripheral blood · multiple standard lipid panels; PUFA profiling subset	No ceramide measurement; ↓ n-3 PUFAs and ↑ n-6:n-3 ratio associated with depression, impulsivity, and aggression	↓ n-3 PUFAs + ↑ n-6:n-3 ratio associated with impulsivity, aggression, and suicidal behavior via serotonergic and inflammatory pathways	Variable confounder control across included studies Limitations: Narrative (not systematic); no ceramide data; mechanistic proposals not directly tested in suicidal populations	Indirect — PUFA-inflammation-serotonin axis as framework for lipid-related suicide risk; ceramide extrapolation requires dedicated studies
Ochi et al. (2023)	Cross-sectional case-control; mitochondrial and telomere biology	Adolescent MDD with suicidality and abuse history vs. HC; adults Peripheral blood · telomere length (qPCR); mitochondrial DNA copy number; no ceramide platform	Ceramides not measured; ↓ telomere length and ↓ mitochondrial DNA copy number strongly associated with suicidality and abuse history in adolescent MDD	Suicidality and abuse history independently associated with shorter telomeres and lower mtDNA copy number; mitochondrial dysfunction as biological substrate of suicidal vulnerability	HC included; telomere and mtDNA co-measured; adjusted for key confounders Limitations: no ceramide data; cross-sectional; adolescent sample may not generalize to adults	Indirect/mechanistic — mitochondrial dysfunction as convergent substrate linking ceramide accumulation, oxidative stress, and suicidal vulnerability; motivates ceramide-mitochondria studies in suicidal MDD.
Jiang et al. (2025)	Meta-analysis + case-control study	Suicidal behavior vs. controls; pooled n NR Peripheral blood + hair · cortisol assay; no ceramide platform	No ceramide measurement	↓ long-term cortisol (hair cortisol) in individuals with suicidal behavior; supports HPA-axis multidimensional risk model	Meta-analytic + case-control combination; hair cortisol as long-term HPA indicator Limitations: no ceramide data; cortisol-suicide relationship complex and context-dependent	Indirect/mechanistic — HPA hypoactivation as component of suicidal vulnerability; relevant to ceramide-HPA interaction hypothesis

### 4.14 Indirect evidence and biological plausibility

Multiple lines of evidence indicate an association between lipid metabolism and suicidality. A large meta-analysis including more than 500,000 participants reported that individuals with suicidal behavior exhibit significantly lower levels of total cholesterol, low-density lipoprotein cholesterol (LDL-C), high-density lipoprotein cholesterol (HDL-C), and triglycerides. These alterations were associated with an increased risk of suicidal ideation, suicide attempts, and completed suicide (Wu et al., 2016) The composition of polyunsaturated fatty acids (PUFAs) has also been implicated. Lower levels of n-3 PUFAs, a higher n-6: n-3 ratio, and overall lipid imbalance have been associated with depression, impulsivity, and aggression. These effects are thought to involve modulation of neuronal membrane microenvironments, serotonergic signaling, and inflammatory pathways (Daray et al., 2018) In a retrospective cohort study of 408 suicide attempts, low total cholesterol levels below 165 mg/dL in women were associated with increased suicide mortality, whereas higher cholesterol levels exerted a protective effect, with hazard ratios ranging from 0.968 to 0.970. These findings suggest that lipoprotein levels may reflect specific biological vulnerability to suicide risk (Park et al., 2024) Together, these results provide biological plausibility for a link between lipid alterations and suicide risk by integrating inflammatory, hormonal, and neuronal signaling mechanisms. They also raise the possibility that circulating ceramides, as components of lipid metabolism, may serve as emerging markers of vulnerability. However, the lack of a valid animal model of suicidal behavior remains a major limitation. Suicide involves factors that are unique to humans, such as intentionality and the concept of will, which are essential to the suicidal act and contribute to its complex philosophical, linguistic, and cultural dimensions. Human language, far from being a transparent medium, emerges from a network of biological and cultural adaptive processes that constrain its expressive capacity (Kirby, 2017). A limitation with direct implications for the clinical assessment of depressive and suicidal symptoms.

### 4.15 Proposed mechanistic model linking ceramides, inflammation, and suicide risk

Lipid metabolism functions not only as a marker of metabolic state but also as an active source of inflammatory signals capable of modulating brain function. This section examines evidence linking lipid alterations to inflammation and suicidal vulnerability in MDD/TRD. Evidence from cholesterol and polyunsaturated fatty acids supports a framework where lipid dysregulation promotes behavioral vulnerability, though direct links to ceramides and suicide remain limited.

Paradoxically, the relationship between lipids and suicidality appears inverse to that typically associated with cardiometabolic risk. A systematic review and meta-analysis of 65 studies (1980–2014; n = 510,392) found that suicidal patients had significantly lower levels of total cholesterol, LDL, and triglycerides compared with non-suicidal patients, and lower TC, HDL, and LDL compared with healthy controls. Low TC was associated with a 112% increase in suicidality risk, including increased risk of both suicide attempt and completion (Wu et al., 2016).

These apparently contradictory findings deserve explicit consideration, as a study in first-episode drug-naive MDD patients found a pro-atherogenic lipid profile in suicide attempters, while a pooled analysis of heterogeneous populations found globally lower lipid levels associated with suicidality risk (Ma et al., 2020; Wu et al., 2016). Differences in sample characteristics, illness stage, outcome definitions, and lipid fractions analyzed likely account for this divergence. Ceramides may offer a more refined biological resolution, as they capture sphingolipid-specific alterations related to inflammatory signaling, apoptosis, and membrane integrity that global lipid metrics do not detect. An exploratory metabolomic study analyzing 307 metabolites in 100 pregnant women identified nominal associations between suicidal ideation and specific ceramide species, including C24:1 and C24:0, though no metabolite survived FDR correction, indicating that these findings remain hypothesis-generating and require replication in larger, adequately powered studies before causal inferences can be drawn (Mitro et al., 2020).

Reductions in total cholesterol and n-3 PUFAs have been associated with dysregulation of toll-like receptor and peroxisome proliferator-activated receptor signaling, elevated cytokine production, and disruption of lipid raft organization, with downstream effects on neurotransmission and synaptic plasticity (Daray et al., 2018). Chronic exposure to stress or depressive states may further amplify this cascade, increasing the likelihood of suicidal ideation and behavior. Overall, this model provides a conceptual framework through which ceramides may connect peripheral lipid and inflammatory alterations with central neural circuits governing mood regulation and impulse control, and it offers a basis for future research and the development of potential risk stratification approaches (see Table 3).

**TABLE 3:** Ceramide pathways and related mechanisms in treatment resistant depression.

Treatment-resistant depression (TRD)						
Author/Year [Ref]	Study Design	Population, Matrix and Platform	Ceramide Findings and Severity Correlation	Treatment Response and Suicidal Ideation	Confounders Controlled and Main Limitations	Biomarker Potential
McIntyre et al. (2023)	Narrative review and consensus (TRD definition, prevalence, management)	Adults with MDD meeting TRD criteria (≥2 failed adequate antidepressant trials) Not applicable	Ceramides not reported	No biomarkers currently approved for patient stratification; reliance on clinical/behavioral measures. ↑ suicide risk noted but not biologically quantified	Not applicable (review/consensus) Limitations: no biomarker data; definitional heterogeneity across staging models limits prevalence estimates	Contextual — establishes unmet need for validated biological markers (including ceramides) for TRD stratification
Pan et al. (2023)	Metabolomics case-control study (treatment-refractory MDD + suicidal ideation)	Adults with treatment-refractory MDD and suicidal ideation vs. HC and non-refractory MDD Plasma and CSF · broad metabolomics platform; purine, ceramide, and energy metabolism panels	Significant alterations in purine metabolism, ceramide-related lipids, and energy metabolites in treatment-refractory MDD with suicidal ideation vs. non-refractory MDD and HC. Ceramide-related metabolic features differentiated TRD + suicidal ideation subgroup	Ceramide and purine metabolic alterations specific to TRD with suicidal ideation; not observed in non-refractory MDD or HC; metabolic signatures may index treatment-refractoriness	Multi-group comparison; plasma and CSF samples; broad metabolomics coverage Limitations: Small n; cross-sectional; cerebrospinal fluid availability limits scalability; ceramide species not individually resolved	High relevance — direct metabolomics evidence linking ceramide-related metabolic features to TRD with suicidal ideation; supports ceramide as a convergent biomarker at the TRD-suicide interface
Kraus et al. (2023)	Observational; GSRD European multicenter database; BMI-stratified	MDD patients from GSRD; n NR Clinical database (anthropometric + clinical) · clinical metabolic variables; no ceramide platform	Ceramides not reported	↑ BMI associated with ↑ suicidality and trend toward higher TRD rates	Multicenter GSRD database; BMI-stratified analyses Limitations: no ceramide or lipid biomarker measurement; retrospective; metabolic confounders not fully controlled	Indirect — adiposity as biological modifier amplifying TRD and suicidality; motivates ceramide-lipidomic studies in overweight/obese TRD.
Schumacher et al. (2022)	Case-control + experimental murine model (see also MDD section)	HC n = 16 vs. untreated MDD n = 16 (mod.–severe); murine chronic stress model Plasma (human); exosomes + hippocampal tissue (mice) · Ceramide sum C16–C24; nSMase2 assays	↑ Plasma ceramides in MDD; nSMase2-dependent ceramide-enriched exosomes inhibit hippocampal endothelial PLD. Neutralization or nSMase2 deficiency prevents depressive behavior HAM-D correlated with ceramide levels	nSMase2 inhibition reverses depressive behavior in mice (therapeutic implication); not assessed in humans (untreated cohort)	ANCOVA: BMI/age/sex; groups comparable on clinical characteristics Limitations: Small human n; aggregated ceramide measure; no longitudinal validation; murine model does not capture full TRD complexity	High mechanistic relevance — nSMase2-ceramide-exosome axis as therapeutic target; nSMase2 inhibition as candidate intervention for ceramide-driven treatment resistance
Brunkhorst-Kanaan et al. (2019)	Observational case-control; lipidomics; multivariable analyses (see also MDD section)	Unipolar/bipolar n = 67 vs. HC n = 405; sex/age/BMI matched Plasma · Targeted + untargeted lipidomics; tandem MS	↑ C16–C24:1 Cer; ↑ C24:1GluCer, C24LacCer Ceramide levels correlated with antidepressant use rather than MADRS improvement	Not associated with MADRS improvement; antidepressant use (not response) predicted higher ceramides	Matching sex/age/BMI; medication status explored as covariate Limitations: Mixed MDD + BD; ceramide elevation pharmacologically driven rather than reflecting treatment response	Medium — ceramides may index pharmacological exposure rather than efficacy; longitudinal studies needed
Rhein et al. (2018)	Experimental pharmacological; *in vitro*/cell-based (two complementary studies)	Cell-based models; no clinical population Cell membrane/lysosomal compartment · ASM activity assays; phospholipidosis quantification	FIASMAs (amitriptyline, imipramine, desipramine, fluoxetine, sertraline, escitalopram, maprotiline) inhibit ASM and reduce ceramide-enriched membrane formation; chemical derivatization enhances ASM inhibition while reducing phospholipidosis	FIASMA activity as mechanism of antidepressant action (indirect); optimized FIASMA derivatives show improved ASM inhibition with reduced phospholipidosis side effects	Controlled *in vitro* conditions; dose-response relationships documented; chemical structure-activity analysis Limitations: In vitro only; clinical relevance of ASM inhibition magnitude requires validation in MDD/TRD patients	High mechanistic relevance — ASM-ceramide axis as pharmacological target; FIASMA chemical optimization strategy for ceramide-targeted antidepressant development
Hoertel et al. (2022)	Observational multicenter; retrospective; COVID-19 hospitalization	Psychiatric disorder patients hospitalized for severe COVID-19; FIASMA-exposed vs. non-exposed; n NR Clinical outcome data · No ceramide platform	No direct ceramide measurement; FIASMA use associated with reduced intubation/death risk	Indirect evidence of ASM-ceramide inhibition as a beneficial mechanism	Multicenter; adjusted for age, sex, comorbidities Limitations: Retrospective; COVID-19-specific context; no ceramide measurement; indication bias possible	Indirect — clinical FIASMA benefit supports ASM-ceramide pathway relevance; motivates prospective TRD trials with ceramide biomarker endpoints
Bose et al. (2025)	Narrative review	Adults with TRD and recurrent MDD; human and animal studies; n varies Peripheral blood + brain · multiple platforms (review)	Ceramides not reported	HPA-TKP interaction influences treatment outcomes; HPA dysfunction → hippocampal atrophy and impaired neuronal plasticity in TRD.	Variable confounder control across reviewed studies Limitations: Narrative review; no primary ceramide data; HPA-ceramide mechanistic link is inferential	Contextual — HPA-ceramide-TKP axis as integrated mechanistic framework for TRD; supports multi-target therapeutic strategies
Sorgdrager et al. (2017)	Observational longitudinal	Recurrent MDD patients + HC; n NR Peripheral blood · cortisol assay; tryptophan/kynurenine metabolites (HPLC or LC-MS)	Ceramides not reported	↑ Evening cortisol → ↓ KYN/TRP ratio in recurrent MDD (HPA-TKP interaction)	HC included for comparison; cortisol and TKP metabolites co-measured Limitations: no ceramide measurement; observational; tryptophan-ceramide link is inferential	Indirect/mechanistic — HPA-TKP dysregulation as metabolic context for ceramide biomarker interpretation; relevant to multi-omic TRD stratification

Abbreviations: AD, Alzheimer’s disease; ASM, acid sphingomyelinase; BD, bipolar disorder; BMI, body mass index; Cer = ceramide; CRP = C-reactive protein; D + S = MDD, with suicide; D−S = MDD, without suicide; FA, fatty acid; FIASMA, functional inhibitor of ASM; GDS, geriatric depression scale; GPL, glycerophospholipid; GluCer = glucosylceramide; HAM-D/HAMD, hamilton depression rating scale; HAMA, hamilton anxiety rating scale; HC, healthy controls; HPA, hypothalamic-pituitary-adrenal; HR, hazard ratio; IBD, inflammatory bowel disease; IL, interleukin; KYN, kynurenine; KYN/TRP, kynurenine/tryptophan ratio; 3-HK, 3-hydroxykynurenine; LacCer = lactosylceramide; LC, long-chain; LC-MS/MS, liquid chromatography–tandem mass spectrometry; LDL-c, low-density lipoprotein cholesterol; MADRS, Montgomery–Åsberg Depression Rating Scale; MDD, major depressive disorder; MRM, multiple reaction monitoring; MS, mass spectrometry; NC, normal cognition; nSMase2 = neutral sphingomyelinase 2; NR, not reported; NS, not significant; OxFA, oxidized fatty acid; PANSS, positive and negative syndrome scale; PLD, phospholipase D; PMI, post-mortem interval; PSC, primary sclerosing cholangitis; PUFA, polyunsaturated fatty acid; TAG, triacylglycerol; TC, total cholesterol; TG, triglycerides; TKP, tryptophan-kynurenine pathway; TLR, toll-like receptor; TNF, tumor necrosis factor; TRD, treatment-resistant depression; TRP, tryptophan; UHPLC, ultra-high-performance liquid chromatography; VLC, very-long-chain.

### 4.16 Potential for risk stratification

Available evidence suggests that specific lipid profiles, including ceramides and cholesterol, may help identify individuals at increased risk of suicide, particularly following a previous attempt. For instance, women with low total cholesterol levels exhibit a higher risk of suicide mortality (Park et al., 2024), and imbalances in the n-6: n-3 PUFA ratio have been associated with increased aggression and impulsivity (Daray et al., 2018).

Integrating lipid-based biomarkers with clinical and psychological assessments could facilitate the classification of patient subgroups, improve monitoring strategies, and support the prioritization of preventive interventions. Moreover, combining lipid markers with hormonal indicators, such as cortisol, and behavioral measures may enable the development of more precise risk assessment panels. In this context, evidence that lower long-term cortisol levels in individuals with suicidal behavior may reflect reduced exposure to chronic stress further supports a multidimensional approach to risk evaluation (Jiang et al., 2025). Taken together, these findings provide preliminary biological plausibility for ceramides as candidates to be explored in future suicide risk stratification research.

### 4.17 Ceramides as potential biomarkers for suicide risk

To establish ceramides as reliable biomarkers of suicide risk, longitudinal studies are needed to evaluate causal relationships and the temporal dynamics of lipid alterations in relation to suicidal ideation and attempts. Replication across diverse populations and cultural contexts is also essential, as suicide risk varies by age, sex, and geographic region (Cai et al., 2021; Souza et al., 2023). The integration of ceramide measures into multi-omic frameworks that combine metabolomic, genetic, and inflammatory data would likely improve predictive accuracy. Together, these steps would be necessary to evaluate whether preliminary findings could eventually support the use of ceramides in monitoring, prevention, or the personalization of therapeutic strategies in individuals at risk of suicide. In parallel, greater standardization of diagnostic approaches is required to support a shared international framework for assessment and implementation.

**FIGURE 4:**
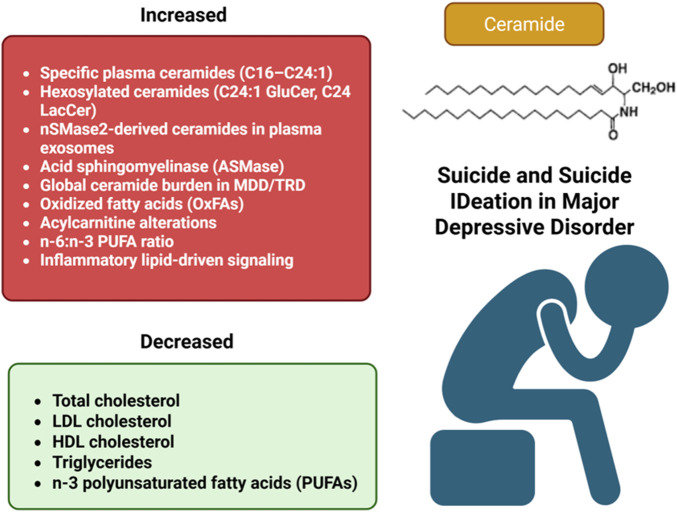
Lipid and ceramide alterations associated with suicidal vulnerability in MDD. Patients with major depressive disorder and treatment-resistant depression exhibit increased levels of specific plasma ceramides (C16–C24:1), hexosylated ceramides, nSMase2-derived ceramides in circulating exosomes, acid sphingomyelinase activity, oxidized fatty acids, and altered acylcarnitines, reflecting enhanced proinflammatory and immunometabolic signaling. In contrast, suicidal vulnerability is associated with reduced total cholesterol, LDL-C, HDL-C, triglycerides, and n-3 polyunsaturated fatty acids, alongside an increased n-6:n-3 PUFA ratio. Together, these lipid alterations suggest a shift toward ceramide-driven inflammatory signaling and membrane dysfunction linked to suicide risk.

As shown in Figure 4, suicidal vulnerability in MDD and TRD is associated with increased circulating ceramides and sphingomyelinase activity, together with reduced cholesterol fractions, triglycerides, and n-3 polyunsaturated fatty acids, suggesting a ceramide-associated immunometabolic and inflammatory lipid profile that warrants investigation as a potential correlate of suicide risk.

## 5 Therapeutic implications

As highlighted throughout this review, lifestyle factors such as diet, physical activity, sleep, and exposure to stress interact directly with the metabolic and inflammatory axes implicated in depression and treatment resistance, including influences related to maternal programming. Maternal depression has been associated with impairments in emotional and behavioral self-regulation in young children. Evidence from studies conducted in Peru and among mothers of Mexican origin indicates that moderate to severe maternal depressive symptoms are linked to reduced regulatory capacity in children, with this association mediated by authoritarian parenting practices (Calzada et al., 2019; Hernández-Vásquez et al., 2022).

Experimental studies in rats further demonstrate that maternal diet during gestation and lactation can induce structural and synaptic alterations in frontomesocorticolimbic circuits of the offspring. These changes involve modifications in AMPA and metabotropic glutamate receptor expression and are associated with the emergence of depression-like behaviors (Trujillo-Villarreal et al., 2021). Collectively, these findings underscore the importance of comprehensive preventive and therapeutic approaches that incorporate healthy lifestyle behaviors and maternal-focused strategies to mitigate emotional and cognitive vulnerability across the lifespan.

### 5.1 Anti-inflammatory strategies with metabolic impact

Therapeutic strategies for MDD and TRD may benefit from interventions that target systemic and central inflammation in close integration with metabolic regulation. Accumulating evidence indicates that chronic inflammatory states and sustained proinflammatory microglial activation impair synaptic plasticity and disrupt HPA axis function, thereby reinforcing a self-perpetuating cycle involving stress exposure, elevated cortisol levels, and lipid dysregulation.

Approaches aimed at inhibiting proinflammatory cytokines, modulating nuclear factor κB signaling, and enhancing antioxidant defenses have shown potential to reduce neuroinflammation, restore microglial homeostasis, and mitigate synaptic dysfunction associated with depressive pathology. In parallel, nutritional strategies, structured physical activity, and pharmacological interventions that normalize triglyceride, cholesterol, and ceramide levels may act synergistically to reduce inflammatory burden and metabolic stress.

This integrative framework emphasizes coordinated peripheral and central modulation of inflammatory processes, acknowledging the bidirectional relationship between immune activation and lipid metabolism. Preclinical evidence raises the possibility that ceramide regulation could represent a relevant mechanistic node within this network, motivating further research into therapeutic approaches aimed at modulating ceramide levels (Marschallinger et al., 2020).

### 5.2 Possible targets in ceramide/sphingolipid pathways

Ceramides represent a central regulatory node linking inflammatory signaling and synaptic plasticity in MDD and TRD. Their metabolic pathways, including *de novo* synthesis, the sphingomyelinase pathway, and salvage mechanisms, provide several potential pharmacological targets. In preclinical models, inhibition of neutral sphingomyelinase 2 has been shown to reduce the release of ceramide-enriched exosomes, thereby preventing depressive-like behaviors and synaptic dysfunction. In parallel, modulation of long-chain ceramide species influences neuronal apoptosis and proinflammatory microglial activation.

Targeting these pathways enables the development of therapeutic strategies that simultaneously attenuate neuroinflammation, regulate lipid metabolism, and support neuronal homeostasis. Moreover, the potential combination of selective ceramide inhibitors with agents that modulate glutamatergic signaling and brain-derived neurotrophic factor pathways supports a multitarget approach acting on convergent mechanisms underlying treatment-resistant (Schumacher et al., 2022).

### 5.3 Combined approaches (anti-inflammatory + immunometabolic)

Treatment-resistant major depressive disorder may benefit from therapeutic strategies that concurrently target inflammatory processes and metabolic dysregulation. Postmortem studies indicate that neuroinflammation and microglial activation interact with alterations in lipid metabolism, including changes in ceramides and triglycerides, creating a self-reinforcing cycle that disrupts synaptic function and neuronal plasticity.

Interventions aimed at reducing proinflammatory microglial activation, together with strategies that modulate circulating lipid profiles, may help restore effective communication between glial cells and neurons and promote neuronal homeostasis. Clinical trials involving cytokine inhibitors, antioxidant therapies, and lifestyle modifications such as dietary changes or increased physical activity suggest that the simultaneous normalization of inflammatory and metabolic pathways may be more effective than targeting a single mechanism in isolation. These findings underscore the importance of combined therapeutic approaches that incorporate both inflammatory and metabolic biomarkers to enhance treatment response in treatment-resistant depression (Li et al., 2025).

### 5.4 Lipid biomarkers to guide treatment and stratified trials

Lipidomic and metabolomic profiling of peripheral blood has enabled the identification of specific ceramide signatures associated with major depressive disorder and other affective conditions. As discussed previously, multiple studies have demonstrated the relevance of distinct lipid profiles in MDD and related disorders.

In one study of 112 adolescents using ultra-performance liquid chromatography–tandem mass spectrometry, several ceramide species, including Cer (18:0/15:0), Cer (18:1/16:0), Cer (18:1/26:0), Cer (18:0/22:0), Cer (d18:0/18:1), and CerP (18:1/18:0), were identified as highly discriminative between healthy controls and patients with PPP and CPP, achieving areas under the curve ranging from 0.938 to 0.964 (Nguyen et al., 2024).

In a separate analysis involving 67 patients with unipolar or bipolar disorder and 405 control participants, combined targeted and untargeted lipidomic approaches revealed elevated levels of C16–C24:1 ceramide and hexosylated metabolites. These alterations were more pronounced in males and correlated with age, triglyceride levels, and antidepressant use, independent of clinical episode status or changes in Montgomery–Åsberg Depression Rating Scale scores ((Brunkhorst-Kanaan et al., 2019).

Among women of Han ethnicity with MDD or bipolar disorder during a depressive episode, lipidomic profiling identified significant alterations in sphingolipids, glycerophospholipids, and acylated fatty acids. In this cohort, 20 lipid species were highlighted as combinational biomarkers for MDD, 8 for bipolar disorder, and 13 for distinguishing between the two conditions, with these profiles correlating with clinical severity as assessed by HAMD, HAMA, and PANSS scales (Zhang et al., 2022).

Finally, a study including 107 patients with MDD and 97 healthy controls reported significant differences in 40 lipid species, notably oxidized fatty acids and acylcarnitines, with oxidized fatty acids showing the strongest discriminative capacity for MDD (He et al., 2025).

Altogether, these findings reinforce the potential of ceramides and other lipid species as peripheral biomarkers of MDD. However, further research is required to clarify their causal role and translational relevance in disease mechanisms.

### 5.5 Personalized medicine perspective in MDD and TRD

Lipidomic profiles, particularly those involving long-chain ceramides and hexosylated metabolites, have shown utility in differentiating major depressive disorder from bipolar disorder and are associated with symptom severity. These alterations appear more pronounced in men, whereas in women, combinations of sphingolipids, glycerophospholipids, and fatty acids more closely reflect clinical severity (Brunkhorst-Kanaan et al., 2019; Zhang et al., 2022) Preclinical evidence indicates that plasma-derived exosomes enriched in ceramides can induce depressive-like behaviors by inhibiting hippocampal phospholipase D activity and reducing phosphatidic acid levels, whereas neutralization of these exosomes prevents such behavioral effects (Schumacher et al., 2022).

These observations support the integration of lipid profiling into personalized medicine approaches aimed at patient stratification and treatment optimization, particularly for therapies targeting AMPA-NMDA signaling, brain-derived neurotrophic factor pathways, and mTOR signaling (Freudenberg et al., 2025). Nevertheless, additional longitudinal and translational studies are required to establish the predictive validity of lipid-based biomarkers in clinical practice.

### 5.6 Standardization and rigorous design for reliable lipidomic biomarkers in MDD and TRD

Lipidomic studies in depression and TRD require rigorous standardization to ensure reliability. Preanalytical variables, including sample type, processing, storage, and extraction, influence ceramide and lipid measurements. Analytical platform differences and clinical factors such as age, sex, episode characteristics, and treatments can confound results. Future studies should use consistent protocols, adequate sample sizes, longitudinal designs, validated scales, and thorough documentation of clinical variables to distinguish disease-related lipid alterations from technical or pharmacological effects and support reliable biomarker identification.

### 5.7 Multi-omics integration and machine learning

Integrating lipidomics with complementary molecular layers, including proteomics, metabolomics, genomics, and transcriptomics, enables a more comprehensive understanding of how metabolic, inflammatory, and neuronal pathways interact in MDD and TRD. Bioinformatic approaches and network-based analyses facilitate the mapping of interactions among genes, proteins, and cellular pathways, while machine learning and artificial intelligence methods support the identification of risk patterns and the prediction of treatment response. Genome-wide association studies, transcriptomic analyses, human induced pluripotent stem cell–derived models, and organoids provide platforms to examine how genetic variation and environmental exposures influence brain circuitry. The integration of multi-omics datasets through co-expression and protein network analyses helps prioritize therapeutic targets and supports the development of personalized strategies grounded in complex molecular profiles (Alam et al., 2022; Hicks et al., 2023).

**FIGURE 5:**
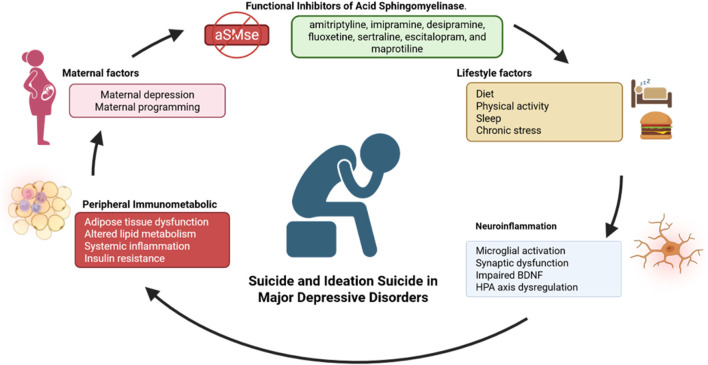
Integrative framework for personalized therapeutic strategies in MDD. This model illustrates the intersection between lifestyle factors, maternal programming, and the immunometabolic axes. Potential pharmacological targets include the inhibition of nSMase2 and the modulation of long-chain ceramide species to restore AMPA/NMDA signaling and BDNF levels. Integration of lipidomic profiling with multi-omics and machine learning facilitates patient stratification, enabling combined anti-inflammatory and metabolic interventions tailored to individual biological signatures to overcome treatment resistance.

The integrative therapeutic framework (Figure 5) highlights the intersection of lifestyle, maternal programming, and the ceramide-nSMase2 axis. Targeted modulation of ceramide species restores AMPA/NMDA plasticity and BDNF levels, while lipidomic profiling enables personalized stratification to overcome treatment resistance in MDD.

## 6 Perspectives and future agenda

Ceramides have been proposed as a plausible mechanistic link between lipid metabolism, peripheral inflammation, and neuronal dysfunction in MDD and TRD, though causal evidence in humans remains limited, with specific species exerting distinct effects on cell death, stress responses, and synaptic plasticity. Circulating and intracellular ceramides may contribute to inflammatory signaling, blood-brain barrier vulnerability, and microglial priming, potentially participating in a cycle that could amplify depressive vulnerability and suicide risk.

Lipidomic profiles differ between MDD and TRD, showing associations with symptom severity, diagnostic subtypes, and sex-specific patterns, with elevated C16–C24:1 ceramide and hexosylated metabolites correlating with higher symptom burden. Despite heterogeneity in human studies, lipid dysregulation has been recurrently observed across studies, though its causal role remains to be established though animal models capture only parts of the human phenotype. Lifestyle, metabolic, and maternal factors, along with comorbidities and pharmacological exposures, influence circulating ceramides and must be accounted for to avoid confounding. Candidate lipid biomarkers include specific ceramides, long-chain and hexosylated metabolites, as well as broader sphingolipids, glycerophospholipids, and altered fatty acid ratios.

These systemic changes accompany synaptic and transcriptomic alterations affecting NMDA and AMPA receptors, BDNF–mTOR signaling, and microglial regulation, alongside HPA axis dysregulation, oxidative stress, and tryptophan–kynurenine pathway imbalances. Collectively, these findings are consistent with a multi-level interplay of lipids, inflammation, and neuronal circuits, and motivate the search for clinically actionable biomarkers. Standardized sample handling, analytical methods, and comprehensive clinical documentation will be necessary to evaluate whether these observations can be translated into risk stratification, longitudinal monitoring, and targeted interventions in MDD and TRD, including potential relevance for suicidal behavior.

Lipidomic evidence is consistent with a role for ceramides as candidate mediators at the intersection of metabolism, inflammation, and synaptic dysfunction, potentially contributing to a cycle that may amplify depressive vulnerability. In men, changes in ceramides are more pronounced, whereas in women combinations of sphingolipids, glycerophospholipids, and fatty acids better reflect symptom severity. Several human studies have reported ceramide patterns associated with depressive symptoms and antidepressant exposure, though findings remain heterogeneous and replication is needed, with variations by sex, age, and comorbidities. Integrating lipidomics with multi-omic data enables differentiation of clinical subtypes and prioritization of therapeutic targets, particularly involving AMPA-NMDA signaling, BDNF, and mTOR pathways.

Future research should standardize sample collection and storage, control clinical and pharmacological confounders, and employ validated clinical scales in adequately powered longitudinal cohorts. Integration of lipidomics with transcriptomics, proteomics, and metabolomics, combined with network-based analyses and machine learning, will allow identification of risk patterns, prediction of treatment response, and distinction between disease-related and treatment-induced changes. Preclinical studies manipulating ceramide metabolism will strengthen causal hypotheses, while longitudinal human studies are essential to validate clinical utility.

Future clinical research should evaluate whether ceramides and hexosylated metabolites can be validated as predictive biomarkers for MDD, TRD, and suicide risk. Multi-omic integration and network analyses may help evaluate whether lipid-based biomarkers can be incorporated into personalized medicine frameworks to inform therapeutic selection and risk stratification.

### 6.1 Limitations of current evidence

Several methodological limitations must be considered. First, lipidomic studies differ widely in platforms used (targeted vs. untargeted), fasting state, and analyte quantification. Second, small sample sizes and lack of replication hinder generalizability. Third, studies often fail to control for confounders such as BMI, metabolic comorbidities, or medication status. Finally, most human studies are cross-sectional, limiting causal inference.

## 7 Conclusion

Ceramides represent biologically plausible immunometabolic mediators linking peripheral lipid dysregulation, neuroinflammation, and neuronal dysfunction in MDD and TRD, with evidence supporting their role in microglial priming, synaptic dysfunction, and blood-brain barrier compromise. Their dysregulation intersects with HPA axis dysfunction, kynurenine pathway imbalances, and mitochondrial deficits, reinforcing depressive vulnerability and treatment resistance. Lipidomic studies consistently report elevated ceramide species in MDD, correlating with symptom severity and sex-specific patterns, supporting their candidacy as biomarkers, though causal evidence in humans remains limited. Current evidence is constrained by cross-sectional designs, small samples, inconsistent confounder control, and heterogeneous analytical platforms, precluding causal inference. Regarding suicidal behavior specifically, the association with ceramide-related signaling is biologically plausible and promising, yet remains empirically unconfirmed, as available evidence is indirect, unreplicated, and methodologically limited. Longitudinal cohorts, multi-omic integration, and standardized protocols are required before ceramide profiles can meaningfully inform risk stratification or therapeutic decisions in precision psychiatry.
