# Supplementary material for: Ceramides and neuroinflammation as immunometabolic drivers and biomarkers of major depressive disorder, treatment-resistant depression, and suicidal vulnerability
Source: Front Pharmacol. 2026 Apr 14;17:1805884. doi: 10.3389/fphar.2026.1805884 (PMC13121294; doi:10.3389/fphar.2026.1805884)
Supplement: Supplementary file 1 [file Table1.docx]

**Supplementary Table S1.** DSM-5 diagnostic criteria for Major Depressive Disorder.

|  | Symptom |
| --- | --- |
| 1 | Depressed mood most of the day |
| 2 | Markedly diminished interest or pleasure in most activities (anhedonia) |
| 3 | Significant weight loss or gain, or decrease or increase in appetite |
| 4 | Insomnia or hypersomnia |
| 5 | Psychomotor agitation or psychomotor retardation (observable by others) |
| 6 | Fatigue or loss of energy |
| 7 | Feelings of worthlessness or excessive or inappropriate guilt |
| 8 | Diminished ability to think or concentrate, or indecisiveness |
| 9 | Recurrent thoughts of death or suicidal ideation |

*Diagnostic and Statistical Manual of Mental Disorders, Fifth Edition, Text Revision (DSM-5-TR)*.

**Supplementary Table S2**. ICD-10 classification of depressive disorders.

|  | **ICD-10** |
| --- | --- |
| Diagnostic framework | Major depressive disorder classified under F32–F33, based primarily on severity and recurrence of depressive episodes |
| Core features of depressive episodes | Persistently low mood, loss of interest or pleasure, reduced energy and activity, impaired concentration and self-esteem, disturbances in sleep and appetite, with fatigue after minimal effort |
| Severity classification | Episodes categorized as mild, moderate, or severe according to symptom burden and degree of functional impairment |
| Mild depressive episode | F32.0; two or three core symptoms with largely preserved daily functioning |
| Moderate depressive episode | F32.1; greater number of symptoms with marked functional impairment |
| Severe depressive episode | F32.2–F32.3; pronounced symptomatology, with or without psychotic features |
| Recurrent depressive disorder | F33; repeated depressive episodes separated by at least two months of remission |
| Exclusion criteria | Absence of a history of manic or hypomanic episodes |
| Remission specifiers | Partial or complete remission, determined clinically based on symptom course and response to treatment |
